# Molecular structure of titania-supported molybdena: *in situ* Raman and FTIR spectroscopy of distinct Mo^VI^O_*x*_ configurations dispersed on titania

**DOI:** 10.1039/d6ra00034g

**Published:** 2026-05-01

**Authors:** Theocharis Kentri, Paraskevas Dimitropoulos, Konstantina Niavi, Eleana Kordouli, Soghomon Boghosian

**Affiliations:** a Department of Chemical Engineering, University of Patras Patras Greece bogosian@chemeng.upatras.gr; b Institute of Chemical Engineering Sciences, FORTH/ICE-HT Patras Greece; c Department of Chemistry, University of Patras Patras Greece; d School of Science and Technology, Hellenic Open University GR-26335 Patras Greece

## Abstract

*In situ* Raman and FTIR spectroscopy, the former complemented by ^18^O/^16^O exchange, are used to unravel the structural and configurational properties of the (MoO_*x*_)_*n*_ phase dispersed on two TiO_2_ polymorphs (anatase and Degussa P25) at the temperature range of 430–120 °C and Mo surface density in the range of 0.5–5 Mo per nm^2^ under oxidative dehydrated conditions. The dispersed (MoO_*x*_)_*n*_ phase supported on titania is heterogeneous; at coverages below *ca.* 1 Mo per nm^2^, isolated species prevail. Under dehydrated conditions, three MoO_*x*_ species occur on titania in either mononuclear or polynuclear form depending on the temperature and coverage: (i) Species-I with a tetrahedral-like mono-oxo configuration, O=Mo(–O–Ti)_3_ with *ν*_Mo=O_ = 996–999 cm^−1^; (ii) Species-II with a pyramidal-like mono-oxo configuration, O=Mo(–O–Ti)_4_ with *ν*_Mo=O_ = 989–993 cm^−1^; and (iii) Species-III with a di-oxo termination configuration with *ν*_s_/*ν*_as_ = 980–983/965–971 cm^−1^. Species-I is formed with the first order of priority and prevails at low coverages (<1 Mo per nm^2^), while its formation ceases at higher coverages. Species-II prevails at coverages of and above 1 Mo per nm^2^, while it constitutes the building unit of the associated polynuclear (MoO_5_)_*n*_ domains at coverages higher than 2 Mo per nm^2^. Temperature cycling in the 430 °C → 250 °C → 175 °C → 120 °C → 430 °C sequence results in a reversible temperature-dependent Species-II ↔ Species-III transformation, mediated by the surface-retained water molecules. Species-II exhibits higher reactivity than Species-I, both with respect to surface-retained water molecules and hydrogen; the latter is judged from its subjection to facile ^18^O/^16^O exchange. These results are important for tuning the configuration of dispersed MoO_*x*_ sites on titania and designing MoO_*x*_/TiO_2_ catalysts at the molecular level.

## Abstract 2

*In situ* Raman and FTIR spectroscopies unravel the structural properties of the (MoO_*x*_)_*n*_ phase dispersed on two TiO_2_ polymorphs (anatase and Degussa P25) at temperatures of 430–120 °C and coverages of 0.5–5 Mo per nm^2^ under oxidative dehydrated conditions.

## Graphical abstract

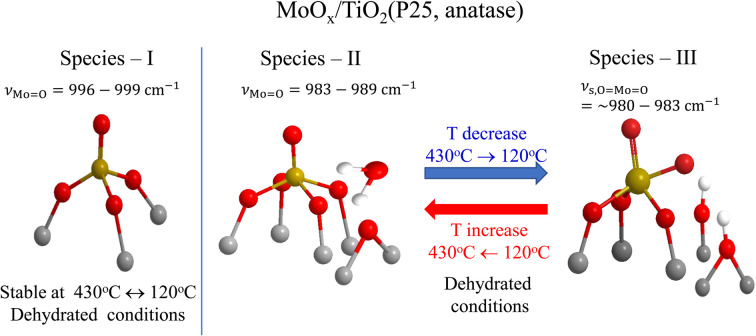

## 1. Introduction

Molybdena dispersed on oxidic supports constitutes an important class of catalytic materials for various processes of industrial and environmental interest and has attracted research interest in recent years.^1–17^ Understanding the structural properties of the (MoO_*x*_)_*n*_ dispersed phase at the molecular level is of paramount importance for deriving structure/function relationships pertaining to supported molybdena catalysts; hence, comprehensive reviews are available on the structural and configurational properties of dispersed (MoO_*x*_)_*n*_^18,19^ and MO_*x*_ (M = V, Mo, W, and Re)^20–31^ overlayers. However, exploring the molecular structure of dispersed molybdena is a formidable challenge due to the occurrence of several species with distinct configurations within the dispersed oxo-Mo^VI^ phase.^7,8^ Studies on SiO_2_-supported molybdena, based on both experimental and theoretical (DFT) evidence, agree that isolated species take on a dioxo configuration for the dispersed MoO_*x*_.^32–42^ To the contrary, there is no consensus for the prevailing domain size and configuration of the (MoO_*x*_)_*n*_ sites on Al_2_O_3_, ZrO_2_ and TiO_2_.^18,19,43–48^ Hence, both isolated (monomeric) and associated (polymeric) species have been reported, primarily possessing a mono-oxo termination configuration with either four-fold (tetrahedral-like) or five-fold (pyramidal-like) coordination for Mo.

Significantly, the typical conditions chosen in experimental *in situ* spectroscopic studies of supported transition metal oxides reflect the so-called oxidative dehydration state, *i.e.* the presence of O_2_(g) to ensure the occurrence of the transition metal in its higher oxidation state and without the presence of H_2_O(g) in the feed gas.^7,8,18–32,49–52^ Additionally, it has often been postulated without sound pertinent experimental evidence that the prevailing MO_*x*_ configurations obtained after the calcination of supported transition metal oxide catalysts are solely driven by thermodynamic constraints irrespective of the synthesis protocol and that they do not revert when temperature is lowered under dehydrated conditions.^20,21,23,25–31,53–57^ However, unprecedented reports demonstrating a reversible temperature dependence of the prevailing species for the dispersed WO_*x*_ and VO_*x*_ phases on TiO_2_ (ref. 7, 8, 58 and 59) as well as dispersed ReO_*x*_ on TiO_2_ and ZrO_2_ (ref. 7, 8, 60 and 61) have controverted the above view, while it has been shown that the synthesis route can affect the speciation of the dispersed MO_*x*_ phase, enabling catalyst design when synthesis methods based on molecular approaches have been followed.^22,62–68^ Despite the underestimation of the heterogeneity issue for the dispersed transition metal oxide phase, *i.e.* the co-existence of distinct species with different structures (*e.g.* pyramidal and tetrahedral) or termination configurations (*e.g.* mono-oxo and di-oxo), several paradigms of dispersed MO_*x*_ phase heterogeneity on oxidic supports have been reported.^2,7,8,69–75^

The comprehensive exploration of the molecular structure and termination configuration of dispersed oxo-metallic phases requires the deployment of the full arsenal of molecular vibrational spectroscopy, including *in situ* Raman and FTIR spectroscopies complemented by ^18^O/^16^O isotope exchange studies.^18,19,29^ The absence of long-range order within the dispersed MO_*x*_ phases makes molecular vibrational spectroscopy particularly suitable for studying the structural properties of the dispersed MO_*x*_ phases. Hence, we take the view that the controversy of pre-2010 studies is to be identified in a number of deficiencies, including one or more of the following shortcomings: (i) applying only one type of vibrational spectroscopic method (*i.e.* either Raman or FTIR) without isotope exchange studies; (ii) overlooking the effect of coverage, *e.g.* by studying one single sample; (iii) omitting to investigate the temperature dependence of the dispersed phase speciation; and (iv) recording spectra after cooling at room temperature.

The heterogeneity of the MoO_*x*_ phase dispersed in titania has recently been ascertained for low-loaded MoO_*x*_/TiO_2_(P25) catalysts.^7^ The aim of the present work is to explore the temperature and coverage effects on the speciation of the dispersed MoO_*x*_ phase by determining the number of species present as well as their relative presence depending on temperature and coverage under dehydrated conditions. To this end, *in situ* molecular vibrational spectroscopies (Raman, FTIR), the latter in the overtone Mo=O stretching region, complemented by *in situ* Raman-^18^O/^16^O isotope exchange studies and Raman spectroscopy under forced dehydrated static equilibrium conditions^8,58,59^ have systematically been deployed. This study is performed using two titania polymorphs as support materials (anatase and Degussa P25) in the temperature range of 430–120 °C and surface coverage of 0.5–5 Mo per nm^2^. Finally, based on the vibrational selection rules and isotope effects, a differentiation between the termination configurations of the prevailing species is undertaken.

## 2. Experimental section

### 2.1 Preparation and texture of MoO_*x*_/TiO_2_ catalysts

Two different TiO_2_ polymorphs were used as support materials: anatase (Alfa Aesar, with a surface area of 127 m^2^ g^−1^ in its pristine form) and industrial Degussa P25 (with a surface area of 49 m^2^ g^−1^ in its pristine form). The latter consists of 80 wt% anatase and 20 wt% rutile, with a corresponding surface analogy of 90% anatase and 10% rutile, and typically consists of 78% anatase, 14% rutile and 8% of an amorphous phase.^76^ Both support materials were subjected to calcination at 480 °C for 4 h prior to catalyst synthesis, which was performed using the wet-impregnation method with (NH_4_)_6_Mo_7_O_24_·4H_2_O (Alfa Aesar, with a metal basis purity of 99.999%) as the molybdena precursor. Two sets of supported catalysts were prepared (each set corresponding to either TiO_2_(anatase) or TiO_2_(P25)), each comprising six samples with sub-monolayer coverage in the 0.5–5 Mo per nm^2^ range, as shown in Table 1. Notably, the reported molybdena monolayer coverage for several oxometallic supports is ∼5 Mo per nm^2^.^19,77,78^ The concentration of the precursor solutions was adjusted in each case to correspond to the desired nominal Mo surface density. During the stage of precursor dissolution and support impregnation that took place at 50 °C under agitation for 1 h, the pH was continuously measured/controlled to pH = 7.5 using either 0.1 M ammonia solution (NH_3_) or diluted nitric acid solution (HNO_3_). In the applied concentration range of 8.4 × 10^−4^–1.6 × 10^−2^ M and for a 7.5 pH value, the oxo-Mo^VI^ species in the solutions and at the solution/titania interface occur exclusively in the form of MoO_4_^2−^, *i.e.* as tetrahedral monomeric species.^65,66^ After the impregnation stage, the solvent was removed by rotary evaporation under reduced pressure at 50 °C. The obtained pastes were dried at 120 °C for 16 h, and the final catalyst samples were obtained after calcination at 480 °C for 4 h under static air in a muffle furnace.

**Table 1:** MoO_*x*_/TiO_2_ catalysts and their specific characteristics (surface density, *n*_s_, in Mo per nm^2^; loading (wt% Mo); BET specific surface area, *S*_BET_, in m^2^ g^−1^; initial concentration of the precursor solution, *C*_Mo(vi)_, in M; and support type). Calcination conditions: 480 °C and 4 h

Catalysts	*n*_s_ (Mo per nm^2^)	Loading (wt% Mo)	*S*_BET_ (m^2^ g^−1^)	*C*_Mo(vi)_ (M)	Support
0.52MoTiO_2_(P25)	0.52	0.4	47	8.4 × 10^−4^	P25
1.3MoTiO_2_(P25)	1.3	0.96	48	2.1 × 10^−3^	P25
1.9MoTiO_2_(P25)	1.9	1.4	48	3 × 10^−3^	P25
2.9MoTiO_2_(P25)	2.9	2.2	49	4.7 × 10^−3^	P25
3.7MoTiO_2_(P25)	3.7	2.9	49	6 × 10^−3^	P25
4.3MoTiO_2_(P25)	4.3	3.4	49	7.2 × 10^−3^	P25
0.55MoTiO_2_(a)	0.55	1.1	123	2.25 × 10^−3^	Anatase
1.1MoTiO_2_(a)	1.1	2.2	120	4.5 × 10^−3^	Anatase
2.1MoTiO_2_(a)	2.1	3.9	117	8.1 × 10^−3^	Anatase
2.7MoTiO_2_(a)	2.7	6.0	115	1.0 × 10^−3^	Anatase
3.7MoTiO_2_(a)	3.7	7.7	101	1.3 × 10^−2^	Anatase
4.9MoTiO_2_(a)	4.9	9.2	100	1.6 × 10^−2^	Anatase

The specific surface area, *S*_BET_, of the calcined catalysts was measured by N_2_ adsorption/desorption as described before.^67^ The coverage for each catalyst sample, *n*_s_, in terms of Mo atoms per nm^2^ (Mo surface density) was calculated from the respective *S*_BET_ and Mo wt%, and the obtained results are shown in Table 1, which compiles the catalyst characteristics. The catalyst samples are denoted as *x*MoTiO_2_(P25) and *x*MoTiO_2_(a), as shown in Table 1, where the prefix *x* stands for the respective Mo surface density in Mo per nm^2^, which is calculated using the formula as follows:
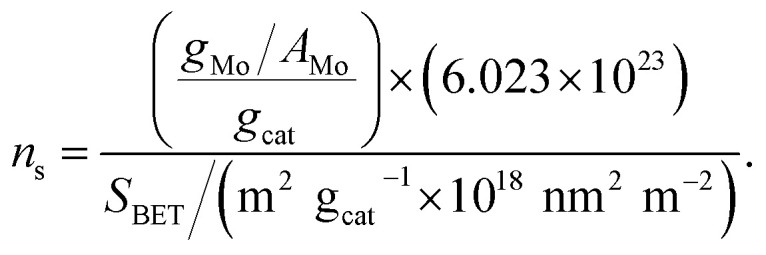


### 2.2 Raman spectra and measurement protocols

#### 2.2.1 Raman spectroscopy under *in situ* oxidative dehydrated feed conditions

A Raman optical homemade furnace was used as the Raman cell for recording *in situ* steady state Raman spectra under oxidative dehydrated conditions. The *in situ* Raman optical cell has previously been described in detail.^19,79^*In situ* Raman spectra were obtained at temperatures in the range of 120–430 °C. To study each catalyst, 120–150 mg of gently powdered material was pressed into a wafer disc (8 mm in diameter and a thickness of ∼1 mm) in a hydraulic press under a typical ∼25 kN load. The excitation source for obtaining the Raman spectra was the 491.5 nm cyan line of a Cobolt Calypso diode-pumped solid-state laser, which was operated at a power of 10 mW on the sample. The incident laser beam was slightly defocused using a cylindrical lens to avoid excessive irradiance. Scattered light was collected at 90° in a horizontal scattering plane, and light analysis was performed using a 0.85 m Spex 1403 double monochromator. A −20 °C-cooled RCA photomultiplier coupled with the Labspec software was used as the detector.

For each sample, the recording of Raman spectra started at 430 °C after 1 h of treatment under oxidative dehydrated feed conditions, namely 20% O_2_/He at a flow of 30 cm^3^ min^−1^. Helium and oxygen gases (99.999%) were obtained from Linde, and the feed gas mixture was sent through molecular sieve traps to remove eventual traces of humidity. The temperature was subsequently lowered to 250 °C, 175 °C and 120 °C, and *in situ* Raman spectra under flowing 20% O_2_/He were obtained after allowing the sample to attain steady state for 1 h and 30 min at each temperature. The temperatures were chosen to span the well-established temperature window of anatase as a support material (*T* < 480 °C) and an adequate number of values (*i.e.* 430 °C, 250 °C, 175 °C and 120 °C) to study the temperature dependence of the molecular structure. The sample was then heated in the *in situ* cell at 430 °C under flowing 20% O_2_/He, and the reinstatement of its initial structure was verified by recording the *in situ* Raman spectra after 1 h of treatment. The specific sequence of temperatures was not relevant to the reproduction of the Raman spectrum at a certain temperature. Hence, using an alternative sequence of temperatures (*e.g.* 430 °C → 120 °C → 175 °C → 250 °C or 250 °C → 430 °C → 120 °C → 175 °C), the *in situ* Raman spectrum could be reproduced at each temperature. The resolution (spectral slit width) was set to 7 cm^−1^ to enhance the signal, and a slow scanning protocol of 1.2 s photon counting per point in increments of 0.25 cm^−1^ was followed to achieve an adequate signal-to-noise ratio. Hence, a 1.5 h duration was necessary for each recording. Furthermore, to control and maintain the measurement precision within ±0.5 cm^−1^, the emission lines of a Ne lamp were recorded in the spectral region of interest to correct for eventual monochromator drifts. To account for the so-called “path length” effect caused by the Mo surface density variation in the 0.5–5 Mo per nm^2^ range, the obtained Raman spectra were subjected to an earlier described procedure of normalisation.^8,80^

#### 2.2.2 FTIR spectra under *in situ* oxidative dehydration conditions

In order to complement the information on vibrational properties pertaining to the (MoO_*x*_)_*n*_ dispersed on TiO_2_(anatase) and TiO_2_(P25) obtained using *in situ* Raman spectroscopy, *in situ* FTIR spectroscopy was used following the same protocol pertaining to sample exposure and temperatures, *i.e.* at a cooling sequence of 430 °C → 250 °C → 175 °C → 120 °C and back at 430 °C, under flowing 20% O_2_/He and subjection of samples for 1 h and 30 min at each temperature. A Nicolet 6700 FTIR spectrometer equipped with a Spectra Tech DRIFT *in situ* cell was used, possessing an MCTB detector and a KBr beam splitter. Each spectrum was the average of 64 scans at a 4 cm^−1^ resolution. The Mo–O overtone region is exploited due to the strong absorption in the respective wavenumber range of the Mo=O fundamental modes. *In situ* FTIR spectra were also obtained under the same conditions, *i.e.* flowing (30 cm^3^ min^−1^) 20% O_2_/He mixture, for the TiO_2_(anatase) and TiO_2_(P25) support materials at each recording temperature. The spectra obtained for the net supports were subtracted from the counterpart *in situ* FTIR spectra obtained for the samples, thereby resulting *in situ* difference FTIR spectra pertaining, in each case, to the dispersed (MoO_*x*_)_*n*_ phases of the catalyst samples. Notably, the penetration depth and scattering properties depend on the packing density, resulting in different overall intensities. Hence, the spectra obtained for each support material at each temperature had to be scaled to match the spectrum obtained for each MoO_*x*_/TiO_2_ sample at the same temperature to produce a difference corresponding to the dispersed phase alone.

#### 2.2.3 Raman spectra under forced dehydrated conditions in sealed quartz cells at static equilibrium

Studying the temperature dependence of the Raman spectra of MoO_*x*_/TiO_2_ catalysts under forced dehydrated conditions has a twofold objective: first, to show that the temperature-dependent variation of the dispersed MoO_*x*_ phase speciation established by the *in situ* Raman and *in situ* FTIR spectra is verified under forced dehydration and second, to show that the temperature-dependent effects seen in the *in situ* Raman and FTIR studies are not due to presence of H_2_O(g) in the incoming 20% O_2_/He feed gas used in the *in situ* studies.

**Fig. 1:**
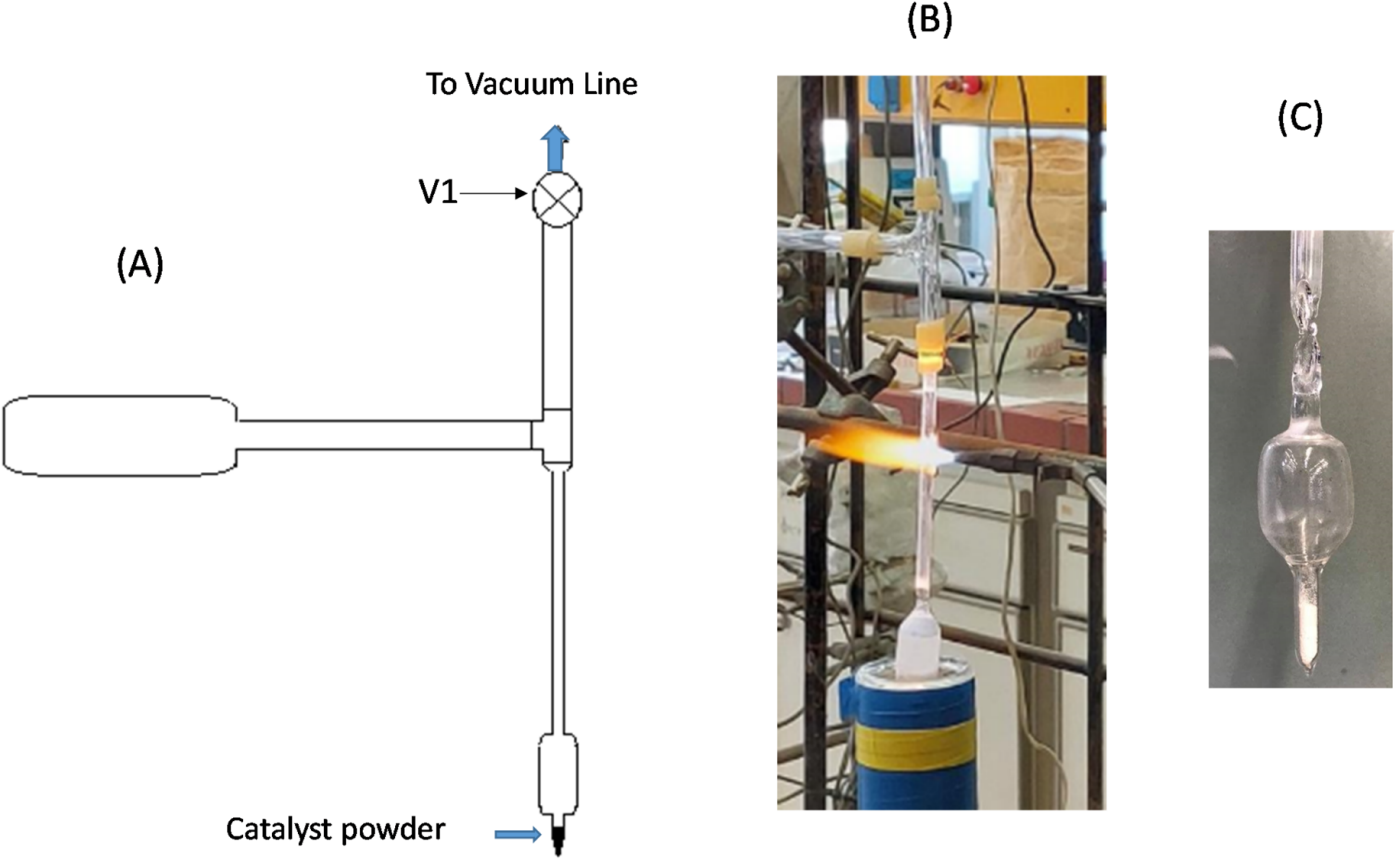
(A) Schematic of the glass assembly used for sealing quartz cells containing catalyst powder under an oxygen atmosphere. (B) Photograph of the quartz cell immersed in liquid nitrogen, for condensing the oxygen gas added in the line, and propane–oxygen torch sealing the cell. (C) Picture of a quartz cell with a bottom appendix containing the catalyst powder and oxygen gas. Reproduced from ref. 59, with permission from the Royal Society of Chemistry.

The concept of static equilibrium Raman measurements in sealed quartz cells has been described elsewhere.^58,59,81^ The quartz cell, shown in Fig. 1, comprises a 2–3 cm long main compartment with 20 mm o.d., a ∼2 cm long appendix (6 mm o.d.) for containing the catalyst powder and a 6 mm o.d. stem. Around 50 mg of each catalyst was added into the cell, which was then attached to a vacuum/gas-addition line. A cylindrical core furnace was then mounted around the cell and heated to 200 °C, and the cell was subjected to dynamic vacuum (∼10^−4^ bar) for 1 h while keeping the trap of the vacuum line immersed in liquid nitrogen to condense any water forcibly removed from the catalyst sample. The valve connecting to the vacuum pump was then closed, and the furnace surrounding the sample was removed. Oxygen gas (Linde, 99.999%) was then admitted to the vacuum line and allowed to condense in the liquid nitrogen trap, thereby establishing an oxygen pressure of *p*_O_2__ = 0.19 bar, *i.e.* the vapor pressure of oxygen at 77 K. Subsequently, oxygen gas contained in a known volume was condensed in the cell appendix by surrounding the cell bottom with liquid nitrogen, and the cell was afterwards sealed with a propane–oxygen torch, as shown in Fig. 1(B). Fig. 1(C) shows an actual photograph of a sealed cell, which has an oxygen pressure of *p*_O_2_,298 K_ = ∼1.0 bar. The ratio of O_2_ molecules to the Mo moles contained in the cell was in the 40–65 range, thereby ensuring that Mo remains in the oxidation state VI. Results are reported from “static” cells made for the representative low coverage 1.3MoTiO_2_(P25) and 1.1MoTiO_2_(a) catalyst samples.

The optical furnace used for obtaining Raman spectra under static equilibrium has been described before.^81,82^ The 532.0 nm green line of a Spectra Physics Excelsior DPSS laser was used as the excitation source, and the scattered light was collected in a 90° horizontal geometry. The laser power was ∼20 mW on the sample. Rayleigh scattering was rejected by an edge filter. The monochromator (with a resolution set at 2 cm^−1^) used was an IHR-320 JY (ISA–Horriba Group) coupled to a CCD detector cooled to −56 °C and interfaced with the Labspec software. Raman spectra were recorded at a sequence of decreasing temperatures in the 430–120 °C range, and the reinstatement of the initial catalyst structure was confirmed by reproducing the Raman spectrum at 430 °C. The quartz cells containing the samples were allowed to reach equilibrium for 1 h and 30 min at each temperature.

#### 2.2.4 *In situ* Raman-^18^O/^16^O isotope exchange

The protocol of the ^18^O/^16^O isotope exchange studies was based on successive reduction/oxidation cycles. During each reduction step, the sample was subjected to a 5% H_2_/He flowing (50 cm^3^ min^−1^) mixture for 30–45 s at 430 °C; the sample was subsequently oxidized under a 2% ^18^O_2_/He flowing (10 cm^3^ min^−1^) mixture for 12 min, and *in situ* Raman spectra were recorded under 2% ^18^O_2_/He after 1, 3, 5, 10, 13 and 18 isotope exchange cycles. The gases used were H_2_ (Linde, 99.999%) and 2% ^18^O_2_/He (Linde, certified). The conditions used (temperature and treatment durations) were sufficient to reach an adequate extent of reduction and a satisfactory ^18^O/^16^O exchange upon reoxidation. The protocol was established using first a 2% ^16^O_2_/He mixture for the reoxidation of the reduced catalysts, as earlier described.^83,84^

## 3. Results and discussion

### 3.1 Configurations of MoO_*x*_ sites dispersed on TiO_2_ (anatase, P25) at low coverage

#### 3.1.1 Temperature dependence of *in situ* vibrational (Raman, FTIR) spectra

**Fig. 2:**
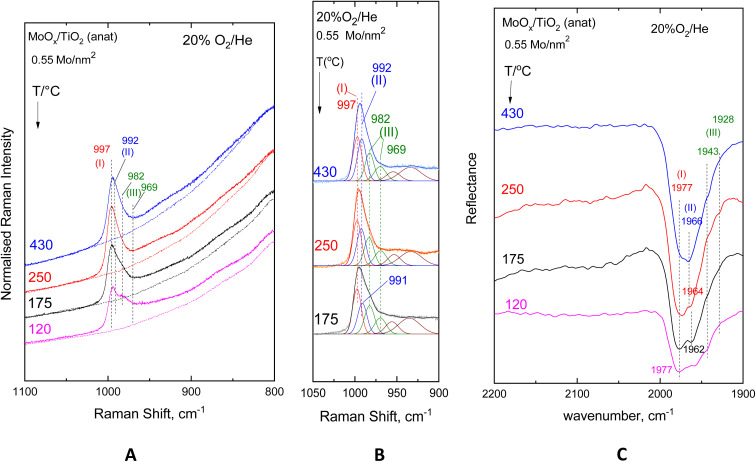
MoO_*x*_/TiO_2_(anatase) with a surface density of 0.55 Mo per nm^2^. (A) Sequential (430 °C → 250 °C → 175 °C → 120 °C) *in situ* Raman spectra obtained under flowing 20% O_2_/He at temperatures indicated by each spectrum. Dotted traces show the corresponding *in situ* Raman spectra of bare TiO_2_(anatase). Laser wavelength, *λ*_0_ = 491.7 nm; laser power, *w* = 10 mW; time constant, *τ* = 1.2 s; spectral slit width, sww = 7 cm^−1^. (B) Peak analysis of the *in situ* Raman spectra shown in panel (A) after subtraction of the TiO_2_(anatase) spectrum at each indicated temperature. (C) Sequential (430 °C → 250 °C → 175 °C → 120 °C) *in situ* FTIR spectra obtained under flowing 20% O_2_/He at the temperatures indicated by each spectrum. Resolution = 4 cm^−1^.

**Fig. 3:**
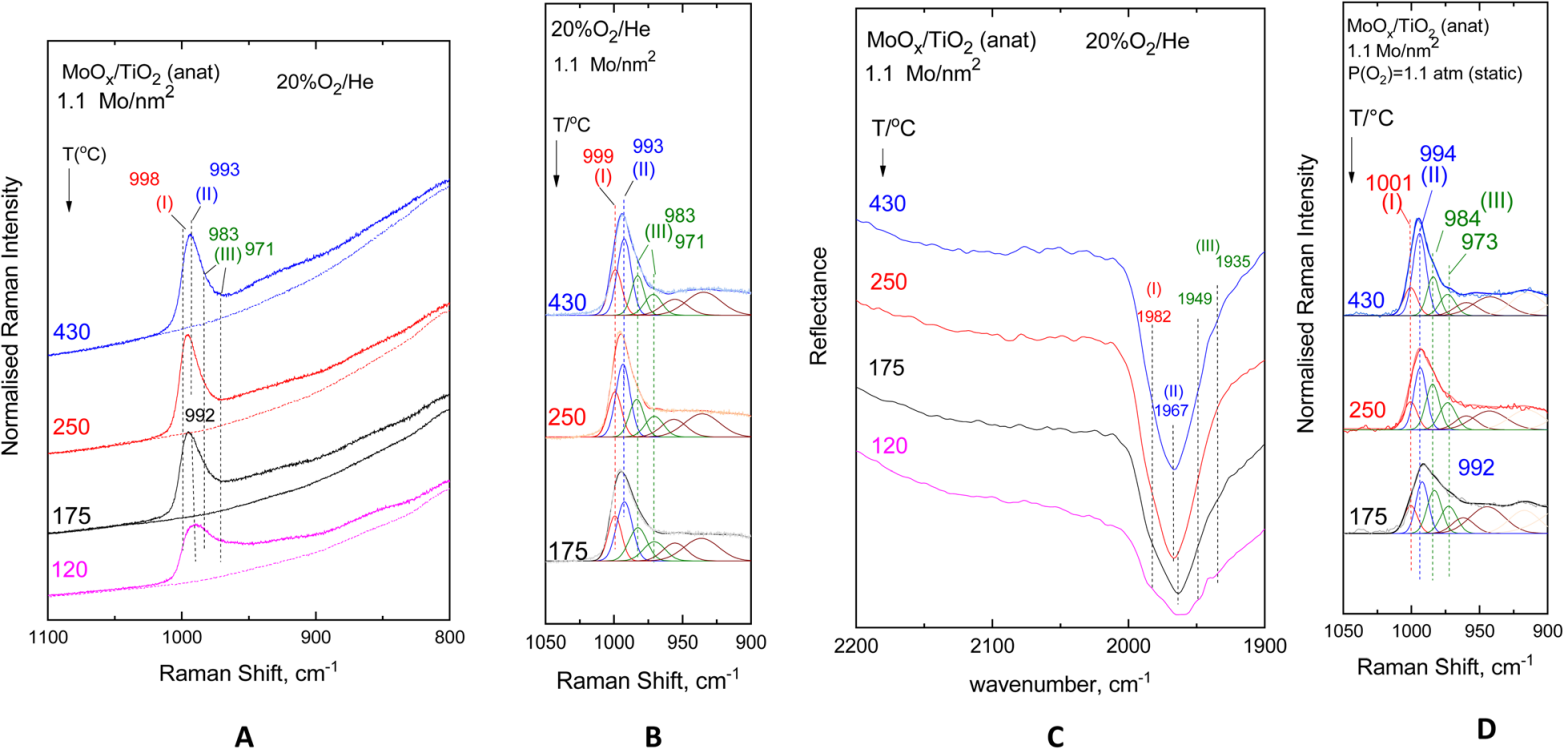
MoO_*x*_/TiO_2_(anatase) with a surface density of 1.1 Mo per nm^2^. (A–C) same as the caption to Fig. 2(D) Peak analysis of the sequential (430 °C → 250 °C → 175 °C) Raman spectra at a static equilibrium under *p*_O_2_,300 K_ = 1.1 atm after subtracting the corresponding spectrum of bare TiO_2_(anatase). Laser wavelength, *λ*_0_ = 532.0 nm, laser power, *w* = 20 mW; and resolution = 2 cm^−1^.

**Fig. 4:**
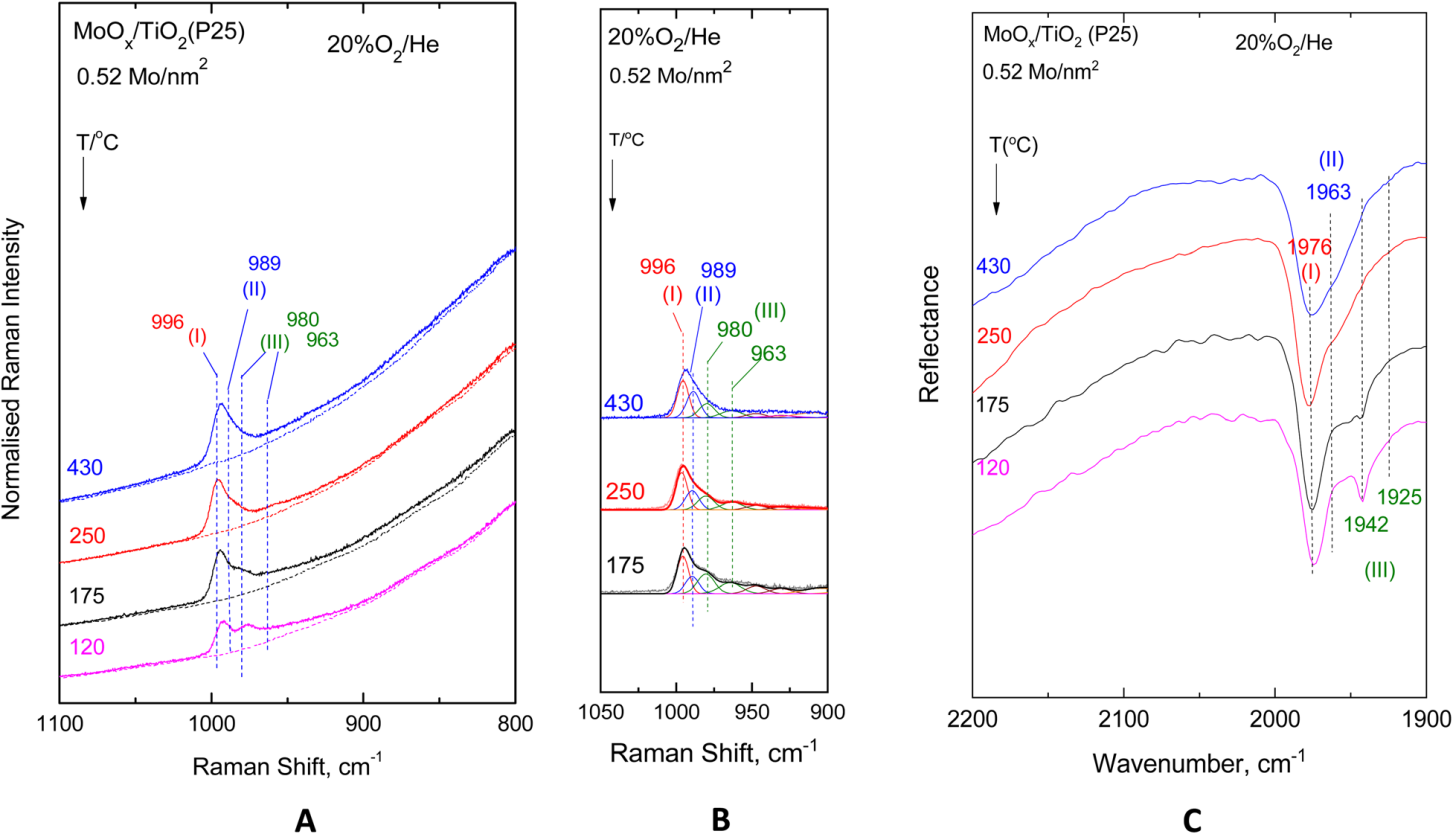
MoO_*x*_/TiO_2_(P25) with a surface density of 0.52 Mo per nm^2^. (A–C) same as the caption to Fig. 2.

**Fig. 5:**
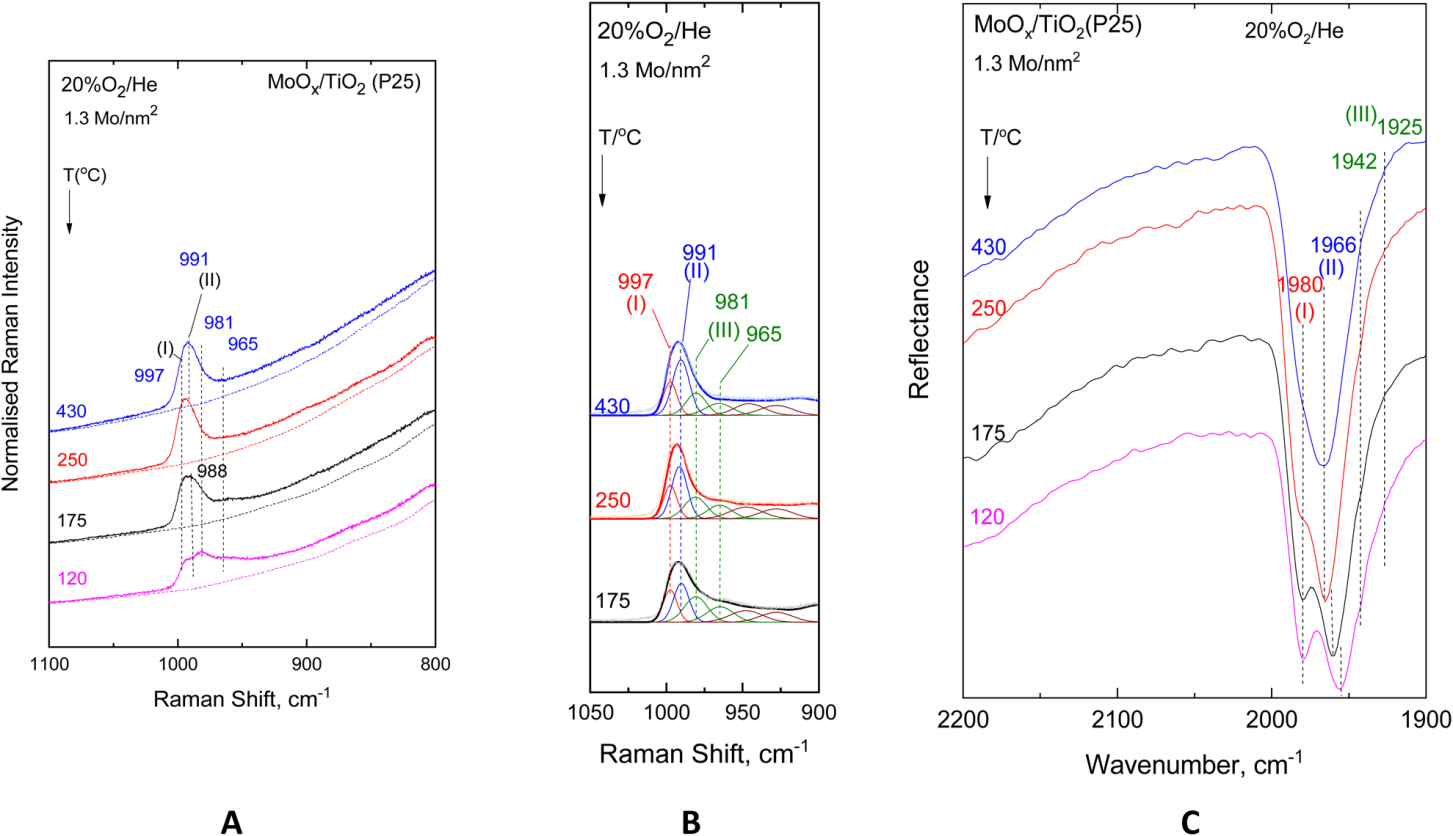
MoO_*x*_/TiO_2_(P25) with a surface density of 0.52 Mo per nm^2^. (A–C) same as the caption to Fig. 2. (D) peak analysis of the sequential (430 °C → 250 °C → 175 °C) Raman spectra at a static equilibrium under *p*_O_2_,300 K_ = 1.3 atm after subtracting the corresponding spectrum of bare TiO_2_(anatase). Recording parameters of the spectra: same as caption to Fig. 3(D).

Fig. 2–5 show the *in situ* vibrational spectra obtained for the low-loaded (0.5–1.3 Mo per nm^2^) Mo/TiO_2_(anatase, P25) samples in the temperature range of 430–120 °C. Notably, the spectra are recorded at a sequence of decreasing temperatures, as described in the Experimental section. Panels (A) portray the temperature dependence of the *in situ* Raman spectra obtained under flowing 20% O_2_/He dehydrated feed conditions. The dotted lines shown under each trace of panels (A) in Fig. 2–5 correspond to the *in situ* Raman spectra of the bare supports, *i.e.* TiO_2_(anatase) or TiO_2_(P25). Panels (B) show the results of peak analyses undertaken in the *in situ* Raman spectra of panels (A) after subtracting the spectra of the bare carriers from the corresponding spectra of the samples in each case. Panels (C) show the temperature dependence of the *in situ* FTIR spectra obtained under flowing 20% O_2_/He, and panels (D) in Fig. 3 and 5 show the peak analysis results undertaken for the Raman spectra obtained under forced dehydration static conditions after subtracting the corresponding spectra of the bare supports.

The Raman spectra, having been subjected to a sound normalization procedure (see Experimental section), provide a consistent set of data for quantitative exploitation. Additionally, the subtraction of the bare TiO_2_(anatase or P25) in each case results in a baseline-corrected net difference spectrum assigned to the dispersed (MoO_*x*_)_*n*_ phase, which is suitable for peak analysis. The fitting was performed using the nonlinear regression method based on the previously described^85^ Levenberg–Marquardt algorithm. The *in situ* FTIR spectra shown in Panels (C) of Fig. 2–5 are used to exploit the relative band intensities that complement the counterpart temperature dependence of the *in situ* Raman spectra.

The Mo=O stretching regions of the *in situ* Raman spectra shown in Fig. 2(A)–5(A) seemingly exhibit at 430 °C a single band feature that appears to possess more than one component, of which the one at the highest wavenumber is denoted as band (I). A second component, denoted as band (II), is discerned in the immediate lower wavenumber position that loses intensity relative to its high wavenumber counterpart band (I) with lowering of the temperature, while at the same time, a third feature (band (III), already existing at an even lower wavenumber but still within the Mo=O stretching region, emerges and grows at the expense of the intermediate wavenumber component, band (II). Lowering of component (II) is discerned in the 430 °C → 250 °C step together with the strengthening of component (III), while at 175 °C and 120 °C, band (II) is obscured by the high wavenumber wing of component (III). Significantly, feature (III) appears to consist of two components, as evidenced by the fact that their mutual relative intensities are maintained constant with varying temperatures. The positions of band (I)–(III) vary slightly depending on coverage and TiO_2_ polymorph and are found based on the peak analysis procedure as follows: 996–999 cm^−1^ for band (I); 989–993 cm^−1^ for band (II); and 980–983/965–971 cm^−1^ for the two components of band (III).

**Fig. 6:**
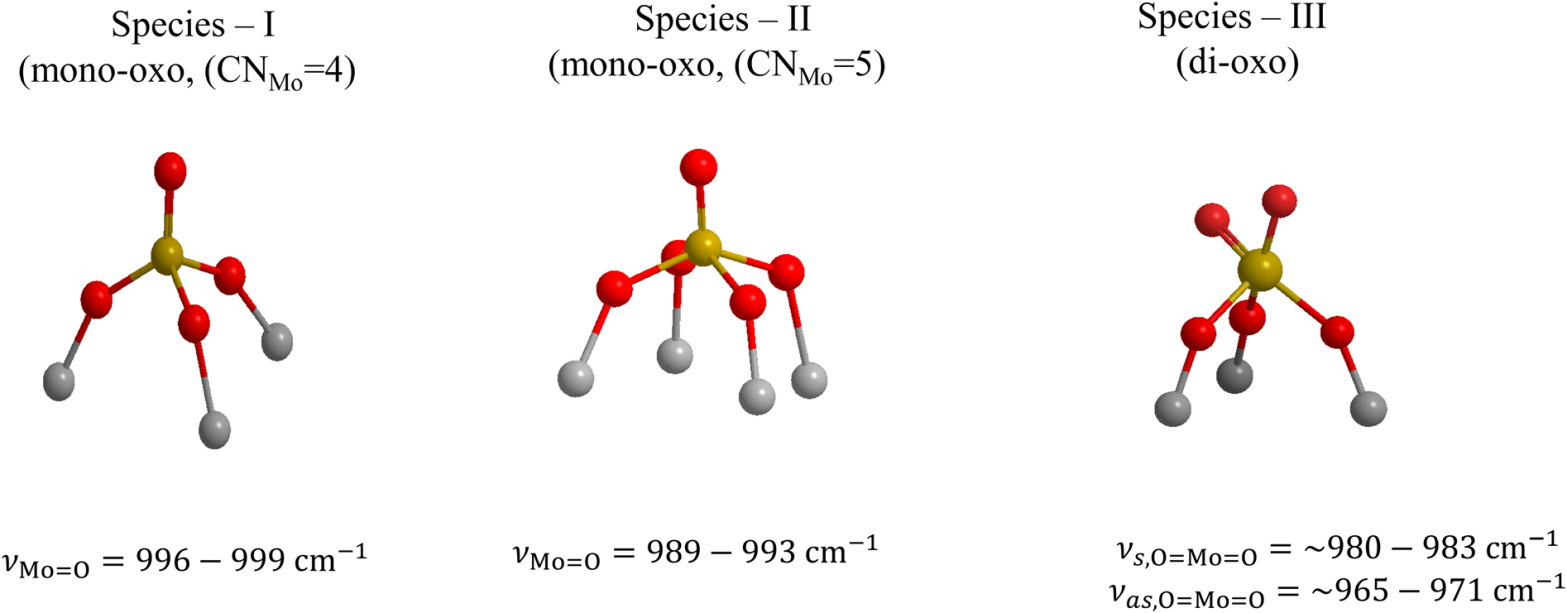
Molecular structural models and Mo coordination/termination characteristics for Species-I, Species-II and Species-III in mononuclear representations. Reproduced from ref. 58, with permission from the Royal Society of Chemistry.

The evidence for the above effects is fully justified and further strengthened by the *in situ* FTIR spectra shown in panels (C) of Fig. 2–5. The *in situ* FTIR spectra are obtained in the first overtone region of the Mo=O stretching modes; hence, not only are the band wavenumbers approximately doubled but most importantly their mutual distances are also doubled, thereby enabling better discernment of the occurrence of the above-mentioned modes and their assignment to discrete species. Notably, the improvement of the separation between the observed bands in the overtone region does not reveal the occurrence of any further bands; hence, it appears that three species are present, of which the relative presence is temperature dependent. When lowering the temperature from 430 °C to 250 °C, band (II) is diminished, thereby allowing for a clear resolution of band (I), while further temperature lowering results in the simultaneous strengthening of the two components of band (III) at the expense of band (II). Hence, bands (I) and (II) are due to distinct sites, each possessing a single terminal Mo=O bond, hereinafter named Species-I and Species-II. Additionally, the double band (III) is due to a site possessing two Mo=O terminal bonds, *i.e.* a di-oxo site, named Species-III. Fig. 6 depicts the proposed structural models for Species-I, Species-II and Species-III in isolated/monomeric form. Species-I and Species-II presumably possess mono–oxo termination configurations, judged from the occurrence of one single Mo=O stretching mode in the vicinity of 1000 cm^−1^,^19^ while Species-III takes on a di-oxo configuration in full conformity with the expected possession of symmetric and antisymmetric stretching modes (*ν*_s_, *ν*_as_) in the lower region of the Mo=O stretching range, *i.e.* below 1000 cm^−1^ at a mutual distance of 10–40 cm^−1^ (*ν*_s_ > *ν*_as_) with the *ν*_s_ mode exhibiting a higher Raman intensity compared to its *ν*_as_ counterpart.^19,86,87^ According to the spectral data displayed in Fig. 2(C)–5(C), the positions of the first overtones of bands (I)–(III) are 1976–1982 cm^−1^ for band (I), 1963–1966 cm^−1^ for band (II), and 1942–1949/1925–1935 cm^−1^ for the two components of band (III).

Previously, both pyramidal-like, with five-fold coordination for Mo(CN_Mo_ = 5), and tetrahedral-like (CN_Mo_ = 4) configurations have been proposed for the mono-oxo O=Mo(–O–Ti)_*x*_ sites dispersed on titania.^7,8,17–21,23,25,30,31,83,88^ The data in Fig. 2–5 strongly suggest the simultaneous occurrence of both types with relative presence controlled by temperature and coverage. In particular, Species-I with its *ν*_Mo=O_ mode at 996–999 cm^−1^ takes on an O=Mo(–O–Ti)_3_ tetrahedral-like mono-oxo configuration, while Species-II with its *ν*_Mo=O_ mode at 989–993 cm^−1^ takes on a O=Mo(–O–Ti)_4_ mono-oxo pyramidal-like arrangement. The wavenumber of a terminal Mo=O stretching mode is not governed solely by the termination configuration (*e.g.* mono-oxo Mo=O and di-oxo Mo(=O)_2_) but also by the Mo coordination number and ligand environment. In the case of the O=Mo^VI^(–O–)_3_ and O=Mo^VI^(–O–)_4_, where the sole difference pertains to the number of oxide ligands, it turns out that, according to the valence sum rule, for a total of 6 v.u., the distribution of bond orders among a large number of bonds results in a lower wavenumber for the Mo=O terminal stretching for O=Mo^VI^(–O–)_4_, where Mo exhibits a five-fold coordination, compared to the respective wavenumber for the O=Mo^VI^(–O–)_3_ species.^79,89^ Hence, the higher wavenumber position for *ν*_Mo=O,Species-I_ compared to *ν*_Mo=O,Species-II_ is fully justified by the lower Mo coordination number for Species-I. As discussed below, at higher Mo coverages, the structural units shown in Fig. 6 occur at adjacent sites and may undergo association (polymerisation) to form larger domains through the formation of Mo–O–Mo and O–Mo–O bridges (*vide infra*). Moreover, with reference to the above documented temperature-dependent Species-II ↔ Species-III interconversion, the following, previously justified,^8,58,59,61^ mechanistic scheme is proposed, accounting for the observed temperature-dependent behaviour of the *in situ* vibrational spectra (Fig. 2–5).
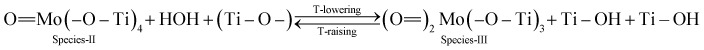


**Fig. 7:**
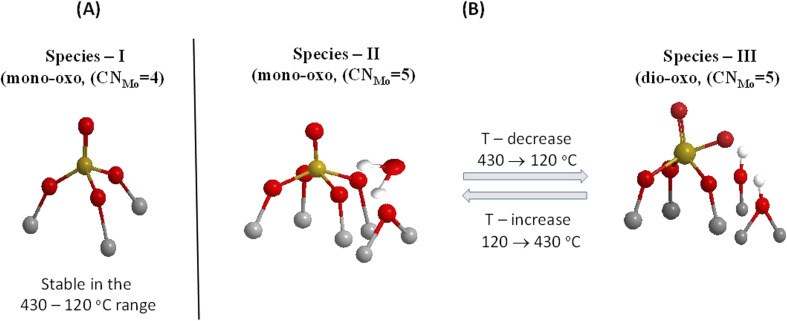
Effect of temperature on the MoO_*x*_ species: (A) Species-I remaining stable in the 430–120 °C range; and (B) mechanism of the Species-II ↔ Species-III temperature-dependent reversible transformation. Reproduced from ref. 58, with permission from the Royal Society of Chemistry.

Fig. 7 portrays the above mechanistic scheme using the proposed molecular models for Species-II and Species-III. Very briefly, the mechanism involves water molecules, which are formed during the impregnation step of the surface hydroxyl titration and retained at the first layer of the support by H-bonds^63,90,91^ that are activated by lowering the temperature, thereby resulting in hydrolysis of anchoring Mo–O–Ti sites and subsequent surface hydroxylation and, in turn, dehydration and dehydroxylation/deprotonation upon heating. This effect is, by nature, expected to occur to a lower extent at high Mo coverage, where fewer surface hydroxyls and vacant deprotonated (Ti–O–) sites are available (*vide infra*).

The wavenumbers of the *ν*_s_/*ν*_as_ pair for a dioxo MoO_2_ core surrounded by O ligands also depend on the coordination number of Mo. Hence, compared to (O=)_2_Mo(–O–Ti)_3_ Species-III (Fig. 6) with CN_Mo_ = 5, the known reference model compounds MoO_2_(SO_4_)_2_^2−^ and MoO_2_(SO_4_)_3_^4−^ possessing MoO_2_ dioxo cores and Mo in six-fold MoO_6_ coordination exhibited their *ν*_s_/*ν*_as_ pairs at 957/918 and 935/895 cm^−1^, respectively,^87^*i.e.* with a wavenumber downshift, justified by the high Mo coordination number.

At this point, it is worth mentioning that the principles and criteria for the peak analysis results shown in Fig. 2–5 panels (B) are as follows:

(i) A minimum number of bands to fit the Mo=O stretching region;

(ii) The occurrence of four bands originating from three species, as evidenced from the *in situ* Raman and FTIR spectra: (a) one band due to Species-I at the highest wavenumber position; (b) one band due to Species-II located at 5–6 cm^−1^ lower compared to its Species-I counterpart; and (c) two bands due to Species-III located at a mutual distance of 10–40 cm^−1^, corresponding to the *ν*_s_/*ν*_as_ pair (with *I*_*ν*_s__ > *I*_*ν*_as__ and fixed *I*_*ν*_s__/*I*_*ν*_as__ ratios, where *I* denotes the band intensity) for a di-oxo species;

(iii) fixed band widths and wavenumber positions (allowing ±1 cm^−1^ for measurement precision) for each sample;

(iv) Larger widths for the di-oxo terminal stretching modes compared to their mono-oxo counterparts.

The validity of the assignments made for bands (I) and (II) can be verified by exploiting the vibrational selection rules of anharmonicity at the diatomic approximation.^86,92^Table 2 compiles the observed band wavenumbers for the *ν*_Mo=O,1←0,R_ Raman fundamentals and *ν*_Mo=O,2←0,IR_ IR first overtones for the mono-oxo Species-I and Species-II as well as the *ν*_s_/*ν*_as_ Raman fundamentals and IR first overtones for the di-oxo Species-III. In the diatomic approximation, the observed vibrational fundamentals and first overtones for the mono-oxo species should comply with the following expressions:

1 *ν*_Mo=O,1←0_ = *ω*_Mo=O_(1 − 2*χ*_Mo=O_),

2 *ν*_Mo=O,2←0_ = 2*ω*_Mo=O_(1 − 3*χ*_Mo=O_),

where *ω*_Mo=O_ and *χ*_Mo=O_ denote the primary (zero-order) wavenumber (corrected for anharmonicity) and the anharmonicity constant, respectively. Hence, the vibrational energy states are not equidistant, and the first overtone is expected at a wavenumber slightly below the doubled observed Raman fundamental,^92^ as is the case with the pertinent data compiled in Table 2. Moreover, the assignments of bands (I) and (II) to mono-oxo terminal stretches in the fundamental and first overtone regions, respectively, of the *in situ* Raman and FTIR spectra are confirmed by the reasonable calculated values for *χ*_Mo=O_ (0.007–0.009, see Table 2) based on eqn (1) and (2).

**Table 2:** Band wavenumbers and their tentative assignments for the Raman fundamentals and IR first overtones of the Mo–O stretching modes of Species-I, Species-II and Species-III

Sample	Species-I, mono-oxo O=Mo(–O–Ti)_3_			Species-II, mono-oxo O=Mo(–O–Ti)_4_			Species-III, di-oxo (O=)_2_Mo(–O–Ti)_3_				O–Re–O
	*ν*_1←0, R_	*ν*_2←0, IR_	*χ*_Mo=O_ a	*ν*_1←0, R_	*ν*_2←0, IR_	*χ*_Mo=O_ a	*ν*_s,1←0, R_	*ν*_as,1←0, R_	*ν*_s,2←0, IR_	*ν*_as,2←0, IR_	
0.55MoTiO_2_(a)	997	1977	0.008	992	1966	0.009	982	969	1942	1928	∼925
1.1MoTiO_2_(a)	999	1982	0.008	993	1967	0.009	983	971	1949	1935	∼930
0.52MoTiO_2_(P25)	996	1976	0.008	989	1963	0.007	980	963	1942	∼1925	—
1.3MoTiO_2_(P25)	997	1980	0.007	991	1966	0.008	981	965	1942	∼1925	∼925

a anharmonicity constants, calculated based on eqn (1) and (2).

The *in situ* Raman and FTIR results for the low coverage MoO_*x*_/TiO_2_(anatase, P25) catalysts in the 0.5–1.3 Mo per nm^2^ range are adequate for a preliminary discussion of the coverage effect. Significantly, vibrational spectra of low coverage catalysts do not “suffer” from the effects of vibrational coupling because extant MoO_*x*_ sites occur primarily in isolated forms, thereby resulting in quite clear spectroscopic signatures pertaining to distinct configurations. Hence, the data in Fig. 2–5 are adequate to suggest that with increasing coverage from ∼0.5 to ∼1.2 Mo per nm^2^, Species-I presence remains stable, while Species-II presence significantly increases. Notably, at very low coverage of ∼0.5 Mo per nm^2^, O=Mo(–O–Ti)_3_ (Species-I) and O=Mo(–O–Ti)_4_ (Species-II) occur to comparable extents on anatase (Fig. 2), while Species-I prevails over Species-II on P25 (Fig. 4). When the coverage is increased to 1.1–1.3 Mo per nm^2^, Species-II clearly prevails over Species-I on both titania polymorphs. It is thus evident that the formation of Species-I occurs in the initial stage of the impregnation step through the titration of most basic hydroxyl sites. Once the most basic hydroxyl receptors are titrated, the formation of Species-II follows. This is in agreement with the higher abundance of high basicity surface hydroxyls on P25 compared to anatase.^58,91^ Moreover, as already mentioned, it is evident that Species-II is favoured at high temperatures, while with temperature lowering, Species-II transforms to Species-III, as shown in Fig. 7.

While at low Mo coverage (*i.e.* ≤1.3 Mo per nm^2^) the majority of the dispersed MoO_*x*_ sites occur in isolated/monomeric form, formation of associated/polymeric (MoO_*x*_)_*n*_ sites is also observed and in fact to different extents on the two TiO_2_ polymorphs. Hence, the broad features observed in the 850–950 cm^−1^ regions in the *in situ* Raman spectra shown in panels (A) of Fig. 2–5 are due to Mo–O–Mo and/or O–Mo–O functionalities.^19,25,30,83,93^ Evidently, a variation of related configurations involving Mo–O–Mo bridges within the dispersed amorphous (MoO_*x*_)_*n*_ phase gives rise to multiple overlapping O–Mo–O and Mo–O–Mo functionalities and vibrational couplings thereof, resulting in a broad continuum in the pertinent region of the Raman spectra. Interestingly, a lower extent of association is evidenced for the TiO_2_(P25)–supported catalysts, as evidenced by comparing the *in situ* Raman spectra shown in Fig. 1(A) and (B) pertaining to the 0.55 Mo per nm^2^ MoO_*x*_/TiO_2_(a) with the corresponding *in situ* Raman spectra shown in Fig. 3(A) and (B) pertaining to the 0.52 Mo per nm^2^ MoO_*x*_/TiO_2_(P25) sample. Fig. S1 (SI) shows two examples of superpositions of spectra obtained for bare TiO_2_(anatase) at 175 °C and 250 °C on the corresponding spectra obtained for 1.1 Mo per nm^2^ MoO_*x*_/TiO_2_(anatase), substantiating the occurrence of a broad continuum in the 850–950 cm^−1^ region, which is ascribed to bridging Mo–O–Mo and/or O–Mo–O modes. The effect of coverage is further discussed below, in a separate section (*vide infra*).

#### 3.1.2 Raman spectra under forced dehydration static equilibrium conditions

The Raman study under forced dehydrated static equilibrium conditions in sealed quartz cells corroborates the findings of the *in situ* vibrational spectra, thereby providing evidence that the water molecules involved in the hydrolysis step of anchoring Mo–O–Ti bonds are not contained in the incoming 20% O_2_/He feed gas of the *in situ* studies but are retained on the support surface even after 1 h of evacuation at 200 °C before sealing the cells for the static experiments (see Experimental section). Hence, as illustrated in panels (D) of Fig. 3 and 5, the same temperature-dependent effects observed are similar to the ones of panels (B) that pertain to the *in situ* studies, namely with temperature lowering band (I) attributed to Species-I remains stable in intensity, while band (II) attributed to Species-II is attenuated in favour of the double band (III) attributed to Species-III. Significantly, it is widely accepted that water is ubiquitous on titania-supported metal oxide systems and that severe conditions (*e.g.* vacuum) are required to achieve absolute absence of water and a fully dehydrated titania surface.^51,52^ Moreover, it has been demonstrated^18^ that heating to 400 °C is required to completely remove water from MoO_*x*_ phases dispersed on titania. Although dispersed MoO_*x*_ is fully dehydrated at 400 °C, water molecules remain on the titania surface, retained, *e.g.*, by H-bonds, as evidenced previously.^62,63,90,94^ Hence, the results of the static equilibrium forced dehydrated Raman studies fully support the mechanism proposed in Fig. 7.

#### 3.1.3 *In situ* Raman with ^18^O/^16^O isotope exchange: vibrational isotope effects

Isotope exchange experiments efficiently complement the studies of *in situ* vibrational spectroscopy of dispersed metal oxide overlayers.^19,29,93^ Notably, by carefully examining the vibrational isotope effects, such as isotopic splitting patterns and isotopic shifts, one may achieve a differentiation between mono-oxo, di-oxo or tri-oxo configurations and justifiably assign bands due to M(=^18^O)_*n*_ modes.

Based on an assumption for a strictly quadratic potential for the Mo=O site in a diatomic harmonic oscillator approximation, the ^18^O/^16^O substitution on the terminal Mo=^16^O site results in a so-called isotopic ratio given by the following formula:^86,92^3
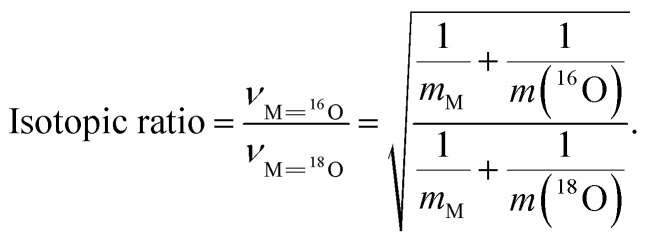


For the Mo=O diatomic harmonic oscillator, the isotopic ratio equals 1.0513.

Assuming the most general quadratic potential functions, the theory of vibrational isotope effects shows that for the symmetric stretching *ν*_1_ and *ν*_2_ modes of a non-linear symmetric XY_2_ molecule (*i.e.* such as the triatomic di-oxo Mo=O_2_ moiety) the 
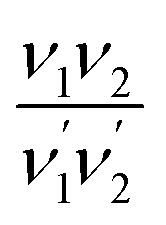
 ratio, where 
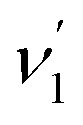
 and 
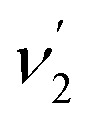
 pertain to the isotopically substituted XY^(i)^_2_ molecule (*e.g.* Mo=^18^O_2_), is independent of the potential constants and of the molecular angle. Moreover, for the case of the di-oxo MoO_2_ unit this ratio can theoretically be calculated equal to 1.052.^95^ Notably, DFT calculations on the (^16^O=)_2_Mo(–^16^O–Si)_2_ → (^18^O=)_2_Mo(–^18^O–Si)_2_ isotopic substitution resulted in a 
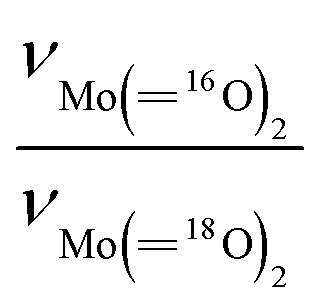
 isotopic ratio of 1.0517,^33^ which is identical to the isotopic ratio of 1.0513, as previewed for the diatomic Mo=O mono-oxo termination, based on eqn (3). Hence, one cannot differentiate between mono-oxo and di-oxo arrangements based solely on the isotopic shift value. In spectroscopically well-documented di-oxo Re(=O)_2_ termination configurations for ReO_*x*_ dispersed on TiO_2_,^60^ CeO_2_ (ref. 74) and ZrO_2_,^61^ it has been shown that the observed isotopic shifts were not adequate to differentiate between mono-oxo and di-oxo termination configurations.

**Fig. 8:**
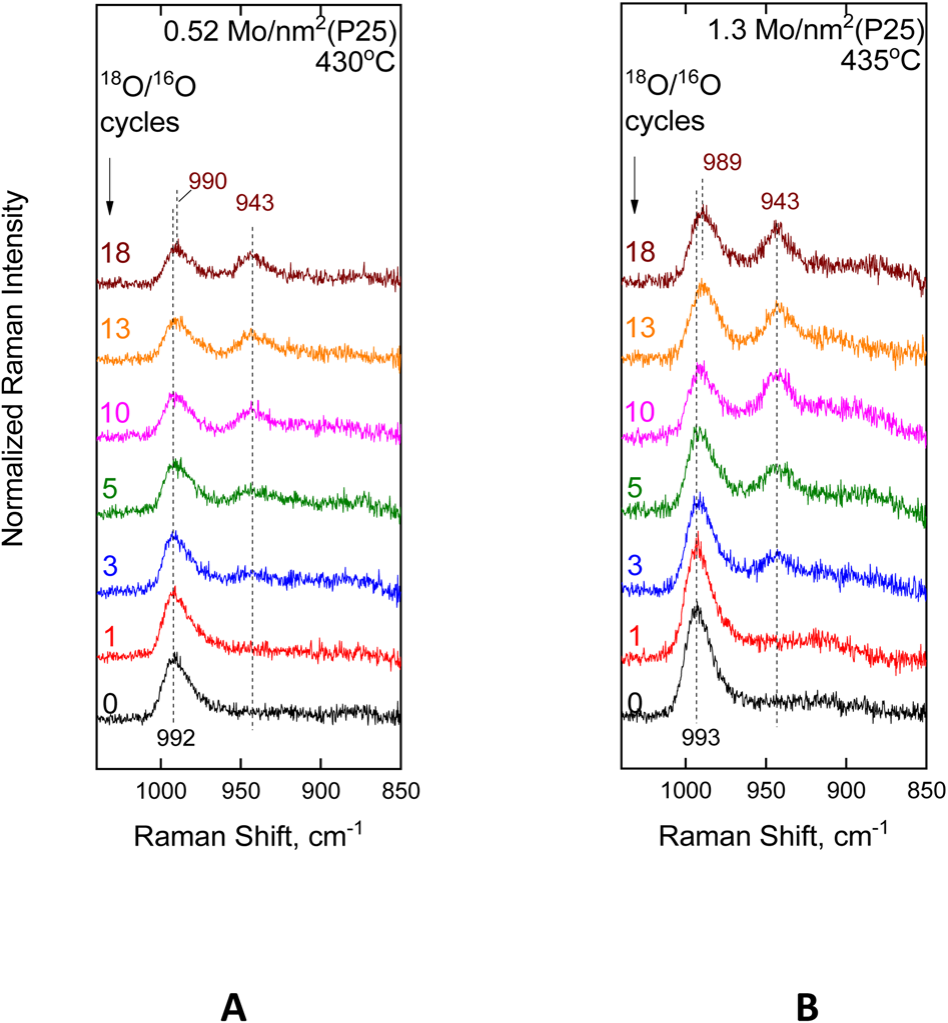
MoO_*x*_/TiO_2_(P25) with surface densities of (A) 0.52 Mo per nm^2^ and (B) 1.3 Mo per nm^2^. *In situ* sequential Raman spectra obtained under flowing 2% ^18^O_2_/He after subsequent H_2_/^18^O_2_ reduction/oxidation isotope substitution cycles, as indicated by each spectrum. Recording parameters: same as the caption to Fig. 2(A).

Fig. 8 shows the sequential *in situ* Raman spectra obtained for the low-coverage 0.52 and 1.3 Mo per nm^2^ TiO_2_(P25)-supported catalyst at 430 °C after ^18^O/^16^O exchange cycles, as indicated by each spectrum. Each ^18^O/^16^O exchange cycle consists of a reduction step under flowing 5% H_2_/He and a re-oxidation step under flowing 2% ^18^O_2_/He (see Experimental section). *In situ* Raman-^18^O/^16^O isotope exchange studies on MoO_*x*_/TiO_2_(anatase) have earlier been reported^83^ and will not be repeated here.

The isotopic splitting pattern for each sample, *i.e.* for 0.52 Mo per nm^2^ in Panel Fig. 8(A) and for 1.3 Mo per nm^2^ in Panel Fig. 8(B), primarily characterizes the termination configuration of the prevailing MoO_*x*_ species in each case, *i.e.* Species-I (O=Mo(–O–Ti)_3_) for 0.52MoTiO_2_(P25) and Species-II (O=Mo(–O–Ti)_4_) for 1.3MoTiO_2_(P25). Notably, in both cases, single splitting is observed, *i.e.* a gradual evolution of one single band due to Mo=^18^O stretching, thereby corroborating the validity of the proposed mono-oxo termination configuration for both Species-I and Species-II. Notably, Species-II undergoes a more facile ^18^O/^16^O exchange of its terminal Mo=^16^O site, as evidenced by comparing the spectral sequences at Panels 8(A) and 8(B) after, *e.g.*, 3, 5 and 10 ^18^O/^16^O exchange cycles.

As previously observed for dispersed O=M(–O–Ti)_*n*_ mono-oxo species (M = Mo, W, and V),^58,59,83^ with an increasing number of ^18^O/^16^O exchange cycles, a gradual red shift is observed for the M=^16^O mode of the non-substituted terminal sites. The observed red shift has been attributed to a gradual ^18^O/^16^O substitution of the anchoring ^16^O atoms that are next-nearest-neighbours to the terminal O atoms,^19,83^*i.e.*^16^O=Mo(–^16^O–Ti)_*n*_ → ^16^O=Mo(–^16^O–Ti)_*n*−1_(–^18^O–Ti) → ^16^O=Mo(–^16^O–Ti)_*n*−2_(–^18^O–Ti)_2_. Importantly, the red shift of Mo=^16^O is clearly discerned from the 3rd ^18^O/^16^O exchange cycle for the 1.3 Mo per nm^2^ sample depicted in Fig. 8(B), thereby pointing to a more facile Mo–^16^O–Ti → Mo–^18^O–Ti exchange for Species-II compared to Fig. 8(A), which pertains to the 0.52 Mo per nm^2^ sample in which Species-I is the prevailing species.

DFT theoretical calculations^44^ showed that dissociative adsorption of hydrogen occurs favourably on unsaturated Ti–O–Ti and deprotonated Ti–O hydroxyl sites, thereby guiding the initial steps of ^18^O/^16^O exchange on lattice O atoms and thereafter by subsequent surface diffusion to anchoring O atoms (Mo–^16^O–Ti → Mo–^18^O–Ti). The lower nuclear charge of ^18^O causes a cascade effect, which weakens the non-substituted Mo=^16^O bond, thereby justifying the observed red shifts in Fig. 8 (992 cm^−1^ → 990 cm^−1^ for 0.52MoTiO_2_(P25) and 993 cm^−1^ → 989 cm^−1^ for 1.3 MoTiO_2_(P25)).

Hence, it turns out that both the terminal and the anchoring O atoms of the pyramidal-like O=Mo(–O–Ti)_4_ Species-II undergo a more facile ^18^O/^16^O exchange compared to the tetrahedral-like O=Mo(–O–Ti)_3_ Species-I.

It should finally be pointed out that the results of the *in situ* Raman-^18^O/^16^O isotope exchange studies on MoO_*x*_/TiO_2_(P25) are in full agreement with the previously reported^83^ pertinent results for the MoO_*x*_/TiO_2_(anatase) counterpart system.

### 3.2 Effect of coverage

**Fig. 9:**
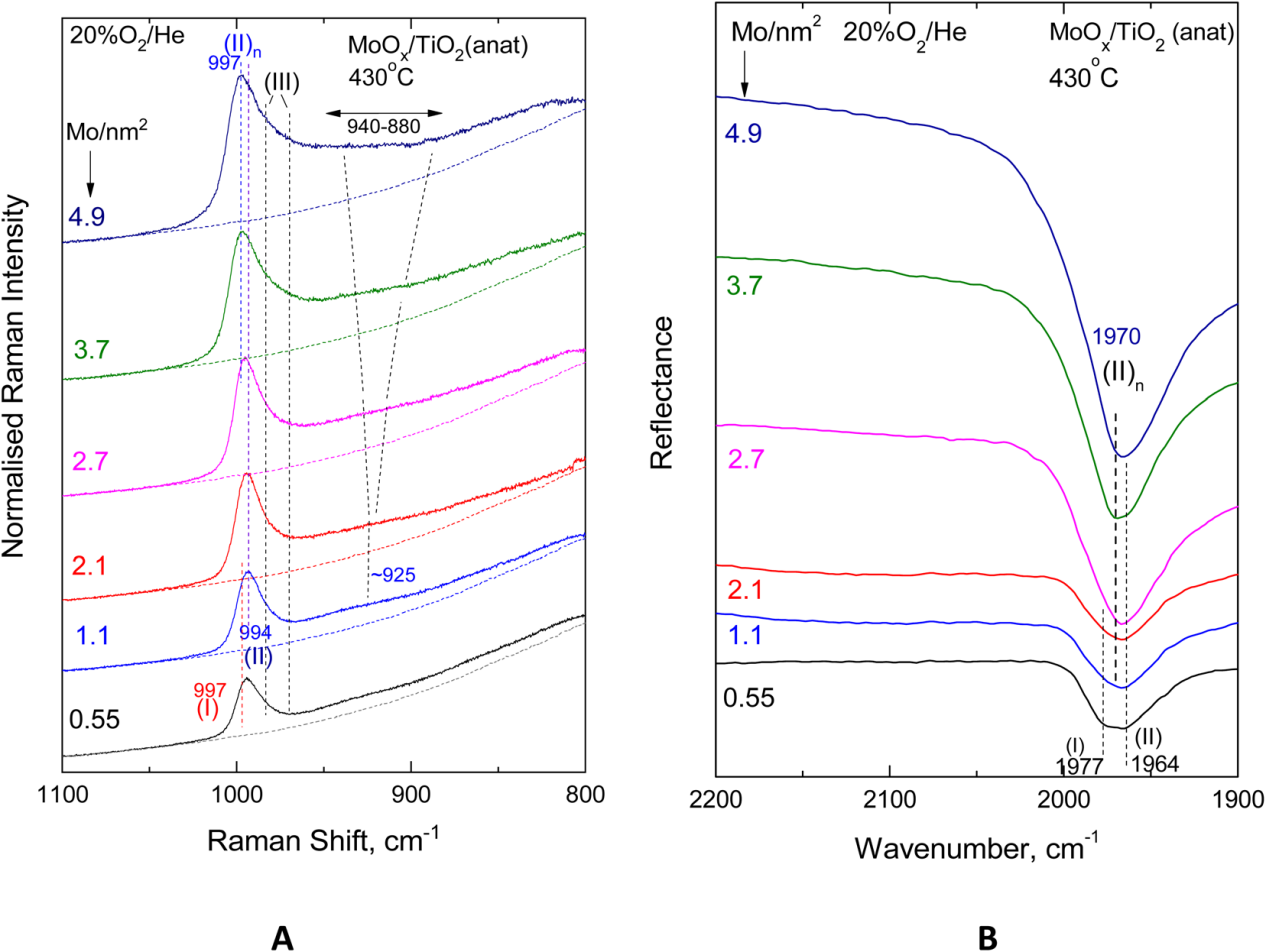
Effect of coverage for the MoO_*x*_/TiO_2_(anatase) catalysts in the 0.55–4.9 Mo per nm^2^ range under flowing 20% O_2_/He at 430 °C. (A) *In situ* Raman spectra. Recording parameters: same as the caption to Fig. 2(A). (B) *In situ* FTIR spectra.

**Fig. 10:**
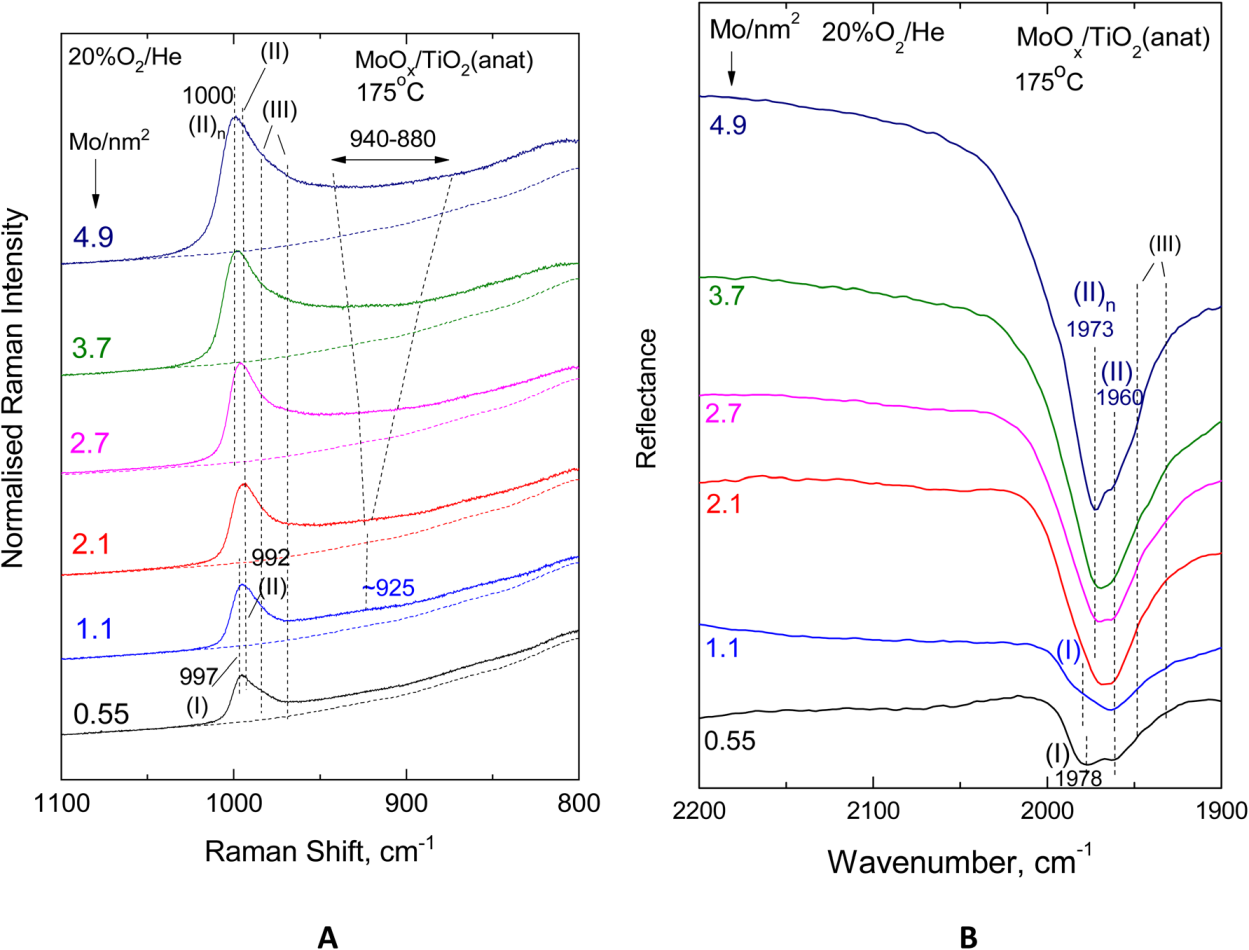
Effect of coverage for the MoO_*x*_/TiO_2_(anatase) catalysts in the 0.55–4.9 Mo per nm^2^ range under flowing 20% O_2_/He at 175 °C. (A) *In situ* Raman spectra. Recording parameters: same as the caption to Fig. 2(A). (B) *In situ* FTIR spectra.

**Fig. 11:**
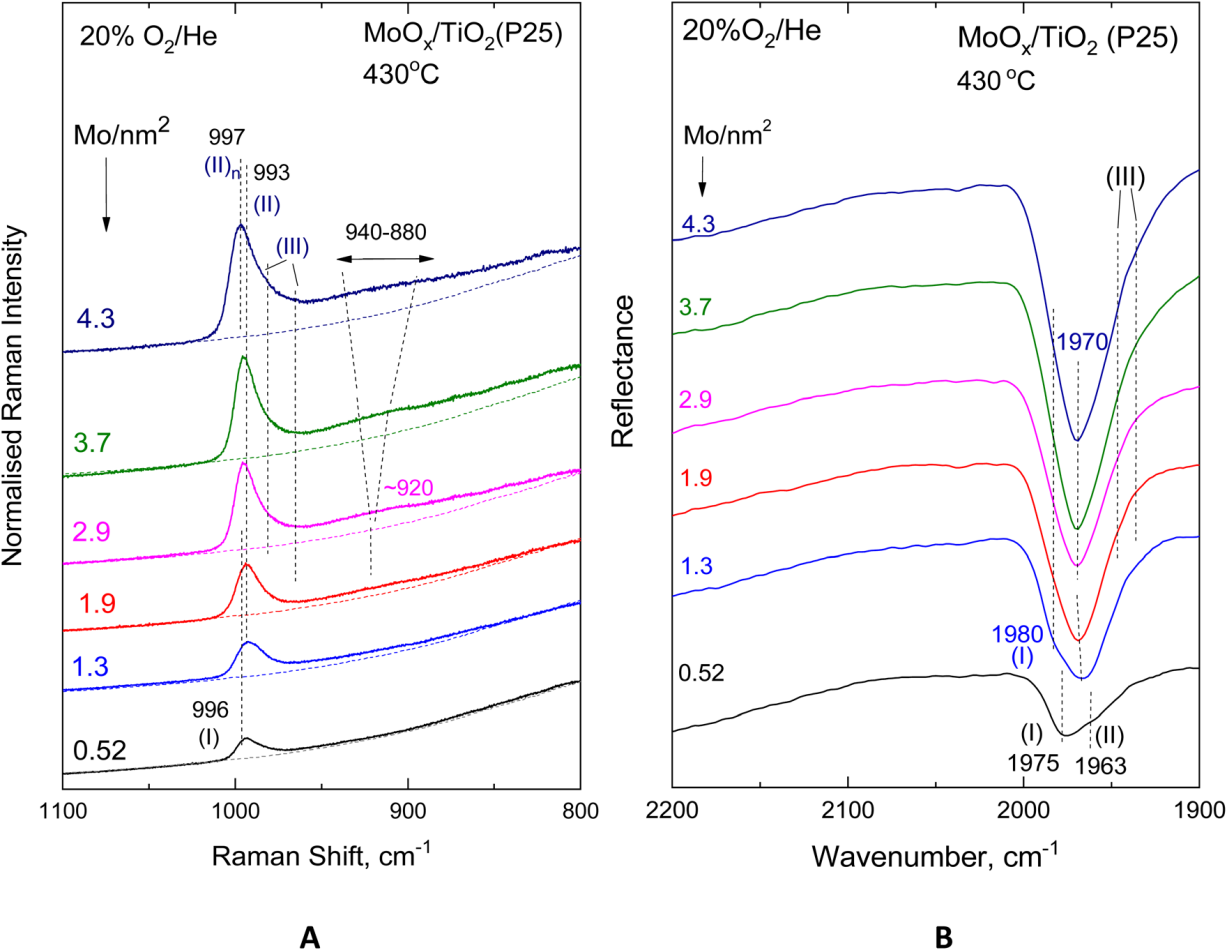
Effect of coverage for the MoO_*x*_/TiO_2_(P25) catalysts in the 0.52–4.3 Mo per nm^2^ range under flowing 20% O_2_/He at 430 °C. (A) *In situ* Raman spectra. Recording parameters: same as the caption to Fig. 2(A). (B) *In situ* FTIR spectra.

**Fig. 12:**
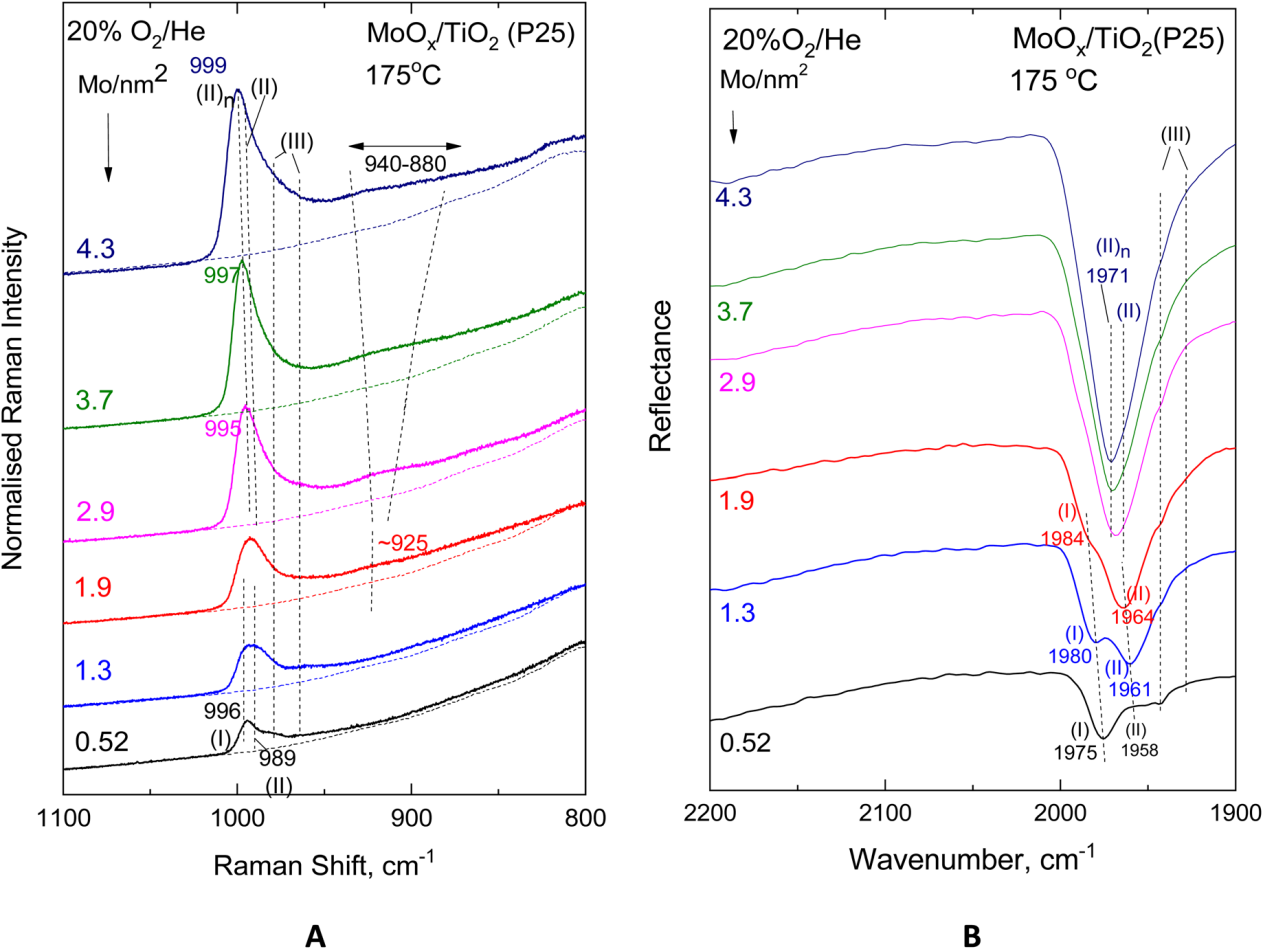
Effect of coverage for the MoO_*x*_/TiO_2_(P25) catalysts in the 0.52–4.3 Mo per nm^2^ range under flowing 20% O_2_/He at 175 °C. (A) *In situ* Raman spectra. Recording parameters: same as the caption to Fig. 2(A). (B) *In situ* FTIR spectra.

Fig. 9 and 10 show the effect of coverage, varied in the range of 0.55–4.9 Mo per nm^2^, in the *in situ* vibrational (Raman, FTIR) spectra obtained for the MoO_*x*_/TiO_2_(anatase) samples at constant temperatures of 430 °C (Fig. 9) and 175 °C (Fig. 10). Likewise, Fig. 11 and 12 portray the effect of coverage, varied in the range of 0.52–4.3 Mo per nm^2^, in the corresponding *in situ* vibrational spectra obtained for the MoO_*x*_/TiO_2_(P25) samples at constant temperatures of 430 °C (Fig. 11) and 175 °C (Fig. 12). All spectra are recorded under flowing 20% O_2_/He. The left panels (panels A) depicted in Fig. 9–12 pertain to *in situ* Raman spectra, while the right panels (panels B) show the counterpart *in situ* FTIR spectra in the overtone region. Fig. S2 and S3 (SI) show the corresponding effects of coverage for the two sets of samples at 250 °C. Notably, the *in situ* FTIR spectra are not used to deduce quantified conclusions when considering spectra of different Mo coverage from different samplings in the same figure panel, *e.g.*Fig. 9(B), 10(B), 11(B) and 12(B), but only to demonstrate differences in the MoO_*x*_ phase speciation for each sample in a qualitative manner. This is due to, as explained in the Experimental section, the dependence of DRIFTS penetration depths on, *e.g.*, sample packing density.

With increasing coverage, band (II) attributed to Species-II grows in intensity and gradually obscures band (I) attributed to Species-I, of which the normalised intensity remains constant, thereby corroborating the observations pertaining to the low coverage samples (*vide ante*, Section 3.1, Fig. 2–5). Hence, the formation of the *tetra*-coordinated mono-oxo O=Mo(–O–Ti)_3_ Species-I precedes the formation of the penta-coordinated mono-oxo O=Mo(–O–Ti)_4_, which continues to form at higher concentrations of the precursor slurries. The gradual prevalence of Species-II over Species-I is best discerned in the *in situ* FTIR spectra (panels Fig. 9(B)–12(B)), where the band separation is nearly doubled in the overtone region. Importantly, the prevalence of Species-II over Species-I with increasing *n*_s_ is also demonstrated for low coverage samples based on the peak analysis results shown in Fig. 2–5 (*vide ante*).

Additionally, with increasing coverage, band (II) attributed to Species-II gradually receives a high-wavenumber component that progressively prevails over its low-wavenumber counterpart, thereby resulting in a blue shift for the convoluted envelope of band (II). Notably, the pertinent blue shifts observed with increasing coverage in the *in situ* FTIR spectra in each case shown in Figure 9(B)–12(B) are approximately double compared to the respective shifts in the corresponding *in situ* Raman spectra in Figure 9(A)–12(A), obtained in the fundamental stretching region. Concomitantly, alongside the blue-shifting tendency of the band (II) envelope, a broad peak mass gradually emerges with increasing coverage in the *in situ* Raman spectra shown in Fig. 9(A)–12(A) and Fig. S2(A) and S3(A). The broad peak mass emerges at surface densities *n*_s_ > 1 Mo per nm^2^ for the samples supported on TiO_2_-anatase (Fig. 9(A) and 10(A)) and at *n*_s_ > 2 Mo per nm^2^ for the TiO_2_(P25)-supported samples (Fig. 11(A) and 12(A)) and gradually expands, seemingly consisting of several contributions, to fill the *ca.* 880–940 cm^−1^ range. The wavenumber location and gradual increase in the broad 880–940 cm^−1^ feature's intensity relative to the terminal Mo=O band intensity with increasing coverage are evident for Mo–O–Mo or O–Mo–O provenance. These bridging modes occur within associated (polymeric) (MoO_*x*_)_*n*_ domains, of which the formation and growth are favoured with increasing coverage and can be subject to vibrational coupling^86^ to each other as well as to anchoring Mo–O–Ti modes, thereby accounting for the observed band overall broadness.

Hence, at low surface densities, *i.e. n*_s_ < 1 Mo per nm^2^, Species-II with penta-coordinated Mo occurs mainly in isolated (monomeric) form, while at high coverages, it constitutes the building unit for the larger associated polymolybdate (MoO_*x*_)_*n*_ domains. The gradual blue shift of the terminal *ν*_Mo=O,Species-II_ mode, observed with increasing coverage (Fig. 9–12), is justified as follows. When the concentration C_Mo(vi)_ in the precursor slurries is increased, more support hydroxyls with progressively lower basicity are gradually titrated during the impregnation step, thereby resulting in lower electron donating abilities of O along Ti–O–Mo anchors and – by cascade effect – to slightly stronger terminal Mo=O bonds.

Previously, in agreement with the present proposed structural properties, density functional theory (DFT) calculations concluded that a distorted tetrahedral *C*_3*ν*_-like mono-oxo arrangement, *i.e.* Species-I, is the prevailing configuration for dispersed molybdena at very low coverage on TiO_2_-anatase.^96^ In particular, Species-I was found to occur on the most stable (101) anatase facet, while di-oxo Species-III was found to occur to a low extent on the minority (001) facet.^96^ Moreover, DFT calculations suggest that with increasing coverage the mono-oxo termination configuration is preserved in species with increased nuclearity (*i.e.* polymeric domains) and that the coordination number of Mo is increased from 4 to 5,^44,97^ in full agreement with the results of the present work. Moreover, experimental L_III_–XANES work on anatase-supported molybdena provided evidence supporting the occurrence of penta-coordinated mono-oxo Mo in polymeric form for samples with high coverage.^77^

Fig. S4–S11 (SI) compile the temperature-dependent features of the sequential *in situ* vibrational (Raman, FTIR) spectra obtained for MoO_*x*_/TiO_2_(anatase) with coverage of 2.1–4.9 Mo per nm^2^ and for MoO_*x*_/TiO_2_(P25) with coverage of 1.9–4.3 Mo per nm^2^ under flowing 20% O_2_/He in the temperature range of 430–120 °C. Notably, the temperature–dependent features therein are *mutatis mutandis* similar to those discussed in the context of Fig. 2–5. Significantly, with increasing coverage, the number of closely related configurations increases, and the bands tend to broaden, overlap and become partially obscured by the growth of the Mo–O–Mo and O–Mo–O modes, whose high wavenumber wing overlaps with the bands attributed to Species-III. Additionally, the occurrence of associated polymolybdate domains gives rise to vibrational coupling, thereby preventing the clear discernment of the spectroscopic signatures due to the distinct extant species.

### 3.3 Implications for reactivity and catalysis

The results of the present study are deemed important for a comprehensive understanding of the structural properties of (MoO_*x*_)_*n*_ sites dispersed on titania polymorphs. Hence, no immediate insight is given to reactivity and catalytic performance, but valuable information is obtained on the constituents of the dispersed phase and on the variation of its speciation depending on temperature and coverage. Moreover, adequate evidence is obtained to infer certain implications for reactivity. The results show that the dispersed (MoO_*x*_)_*n*_ phase is heterogeneous and that the configuration of the prevailing species depends on temperature and coverage. Notably, the catalyst phase, which is active with respect to the catalytic process, may be heterogeneous, while spectator species can also be present. Therefore, knowledge of how to tune the configuration of the prevailing species can improve the efficiency of a catalyst material. Importantly, with respect to reactivity, the results of the present study show that the pyramidal-like O=Mo(–O–Ti)_4_ Species-II exhibits the highest reactivity towards both surface-retained water molecules, transforming to (O=)_2_Mo(–O–Ti)_3_ with temperature lowering, as well as towards hydrogen, thereby undergoing facile ^18^O/^16^O isotope exchange of terminal and anchoring O atoms.

## 4. Conclusions

(a) At elevated temperatures, under oxidative dehydrated conditions, the (MoO_*x*_)_*n*_ phase dispersed on the two titania polymorphs used as supports (anatase, Degussa P25) is heterogeneous. It consists of three distinct building units: (i) Species-I has a mono-oxo tetrahedral-like O=Mo(–O–Ti)_3_ configuration with a *ν*_Mo=O_ terminal stretching mode at 996–999 cm^−1^; (ii) Species-II has a mono-oxo pyramidal-like O=Mo(–O–Ti)_4_ configuration with a *ν*_Mo=O_ at 989–993 cm^−1^; and (iii) Species-III has a di-oxo (O=)_2_Mo(–O–Ti)_*x*_ configuration with a *ν*_s_/*ν*_as_ pair at 980–983/985–971 cm^−1^. The Mo=O terminal stretching wavenumbers depend on coverage, exhibiting a slight blue shift with increasing coverage.

(b) At low coverage, *i.e.* below 1 Mo per nm^2^ for MoO_*x*_/TiO_2_(anatase) and below 2 Mo per nm^2^ for MoO_*x*_/TiO_2_(P25), isolated mononuclear species prevail within the dispersed molybdena phase.

(c) Species-I (O=MoO_3_) prevails over Species-II at a low coverage of below 1 Mo per nm^2^, which is formed with the first order of priority during the impregnation step by the titration of the most basic support hydroxyls. Species-II gradually prevails over Species-I with increasing coverage. Moreover, at coverages exceeding *ca.* 2 Mo per nm^2^, polymolybdate (O=MoO_4_)_*n*_ domains consisting of Species-II building units are predominant.

(d) By lowering the temperature in the 430 °C → 250 °C → 175 °C → 120 °C sequence, the mono-oxo Species-II undergoes a transformation to di-oxo Species-III, which is mediated by water molecules retained at the first layer of the support surface. The temperature-dependent Species-II ↔ Species-III transformation is fully reversible.

(e) Species-I is not affected by temperature cycling in the 430–120 °C range, as evidenced by its fixed *ν*_Mo=O_ mode wavenumber position and stable normalised band intensity.

(f) The pyramidal-like O=Mo(–O–Ti)_4_ Species-II exhibits the highest reactivity towards surface-retained water molecules as well as towards hydrogen, thereby being subjected to the reversible temperature-dependent Species-II ↔ Species-III transformation and to a more facile ^18^O/^16^O exchange compared to Species-I.

(g) These results point to the feasibility of tuning the configurations of the prevailing (MoO_*x*_)_*n*_ sites dispersed on titania polymorphs by the appropriate control of loading and temperature.

## Conflicts of interest

There are no conflicts of interest to declare.

## Supplementary Material

RA-016-D6RA00034G-s001

## Data Availability

The data supporting this work are included in the main article and its supplementary information (SI). Supplementary information is available. See DOI: https://doi.org/10.1039/d6ra00034g.
