# Supplementary material for: Molecular structure of titania-supported molybdena: in situ Raman and FTIR spectroscopy of distinct MoVIOx configurations dispersed on titania
Source: RSC Adv. 2026 May 1;16(25):23019–36. doi: 10.1039/d6ra00034g (PMC13134008; doi:10.1039/d6ra00034g)
Supplement: RA-016-D6RA00034G-s001 [file RA-016-D6RA00034G-s001.pdf]

Supplementary Information (SI) for RSC Advances.
This journal is © The Royal Society of Chemistry 2026

Molecular structure of titania – supported molybdena catalysts: *in situ* Raman and FTIR spectroscopy of distinct $Mo^{VI}O_x$ configurations dispersed on titania

SUPPLEMENTARY INFORMATION

Theocharis Kentri[1,2], Paraskevas Dimitropoulos[1], Konstantina Niavi[1]
Eleana Kordouli[3] and Soghomon Boghosian*[,1,2,4]

[1] *Department of Chemical Engineering, University of Patras, Patras, Greece*

[2] *Institute of Chemical Engineering Sciences, FORTH/ICE-HT, Patras, Greece*

[3] *Department of Chemistry, University of Patras, Patras, Greece*

[4] *School of Science and Technology, Hellenic Open University, GR-26335 Patras, Greece*

(*) to whom correspondence should be addressed

e-mail: bogosian@chemeng.upatras.gr

CONTENTS

- **Figure S1.** Superposition of *in situ* Raman spectra obtained for bare TiO_2(anatase) with the corresponding spectra obtained for 1.1 Mo/nm^2 MoO_x/TiO_2(anatase) under flowing 20% O_2/He at (A) 175 ^oC; (B) 250 ^oC. Recording parameters: Laser wavelength, λ_0 = 491.7 nm; laser power, w = 10 mW; time constant, τ =0.3 s; spectral slit width, sww = 7 cm^{-1}.
- **Figure S2.** Effect of coverage for MoO_x/TiO_2(anatase) catalysts, in the 0.55 – 4.9 Mo/nm^2 range under flowing 20% O_2/He at 250 ^oC. (A) *in situ* Raman spectra. Recording parameters: see the caption to Fig. 2(A). (B) *in situ* FTIR spectra.
- **Figure S3.** Effect of coverage for MoO_x/TiO_2(P25) catalysts, in the 0.55 – 4.9 Mo/nm^2 range under flowing 20% O_2/He at 250 ^oC. (A) *in situ* Raman spectra. Recording parameters: see the caption to Fig. 2(A). (B) *in situ* FTIR spectra.
- **Figure S4.** MoO_x/TiO_2(anatase) with surface density of 2.1 Mo/nm^2. (A) See caption to Fig. 2(A). (B) See caption to Figure 2(C).
- **Figure S5.** MoO_x/TiO_2(P25) with surface density of 1.9 Mo/nm^2. (A) See caption to Fig. 2(A). (B) See caption to Figure 2(C).
- **Figure S6.** MoO_x/TiO_2(anatase) with surface density of 2.7 Mo/nm^2. (A) See caption to Fig. 2(A). (B) See caption to Figure 2(C).

- **Figure S7.** MoO_x/TiO_2(P25) with surface density of 2.9 Mo/nm^2. (A) See caption to Fig. 2(A). (B) See caption to Figure 2(C).
- **Figure S8.** MoO_x/TiO_2(anatase) with surface density of 3.7 Mo/nm^2. (A) See caption to Fig. 2(A). (B) See caption to Figure 2(C).
- **Figure S9.** MoO_x/TiO_2(P25) with surface density of 3.7 Mo/nm^2. (A) See caption to Fig. 2(A). (B) See caption to Figure 2(C).
- **Figure S10.** MoO_x/TiO_2(anatase) with surface density of 4.9 Mo/nm^2. (A) See caption to Fig. 2(A). (B) See caption to Figure 2(C).
- **Figure S11.** MoO_x/TiO_2(P25) with surface density of 4.3 Mo/nm^2. (A) See caption to Fig. 2(A). (B) See caption to Figure 2(C).

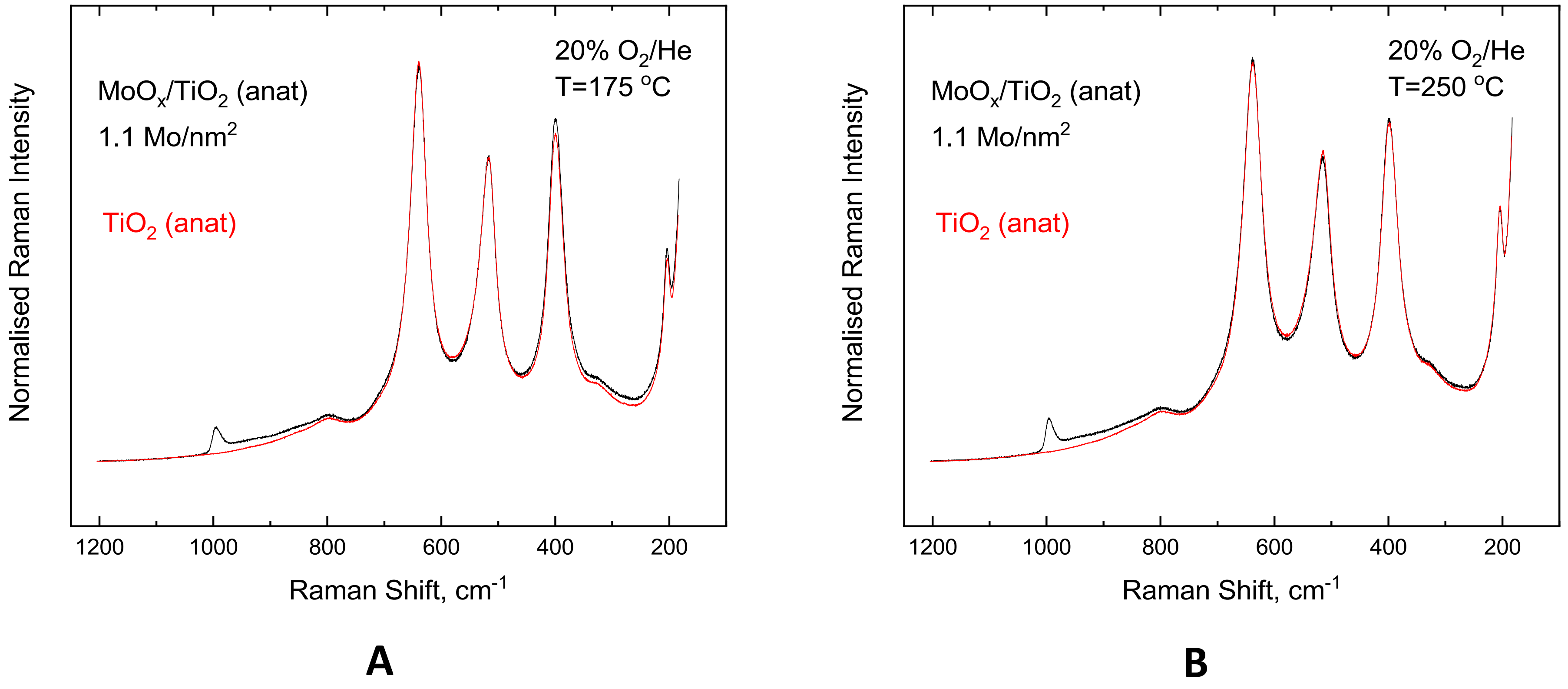

Figure S1. Superposition of *in situ* Raman spectra obtained for bare TiO_2(anatase) with the corresponding spectra obtained for 1.1 Mo/nm^2 MoO_x/TiO_2(anatase) under flowing 20% O_2/He at (A) 175 ^oC; (B) 250 ^oC. Recording parameters: Laser wavelength, λ_0 = 491.7 nm; laser power, w = 10 mW; time constant, τ =0.3 s; spectral slit width, sww = 7 cm^{-1}.

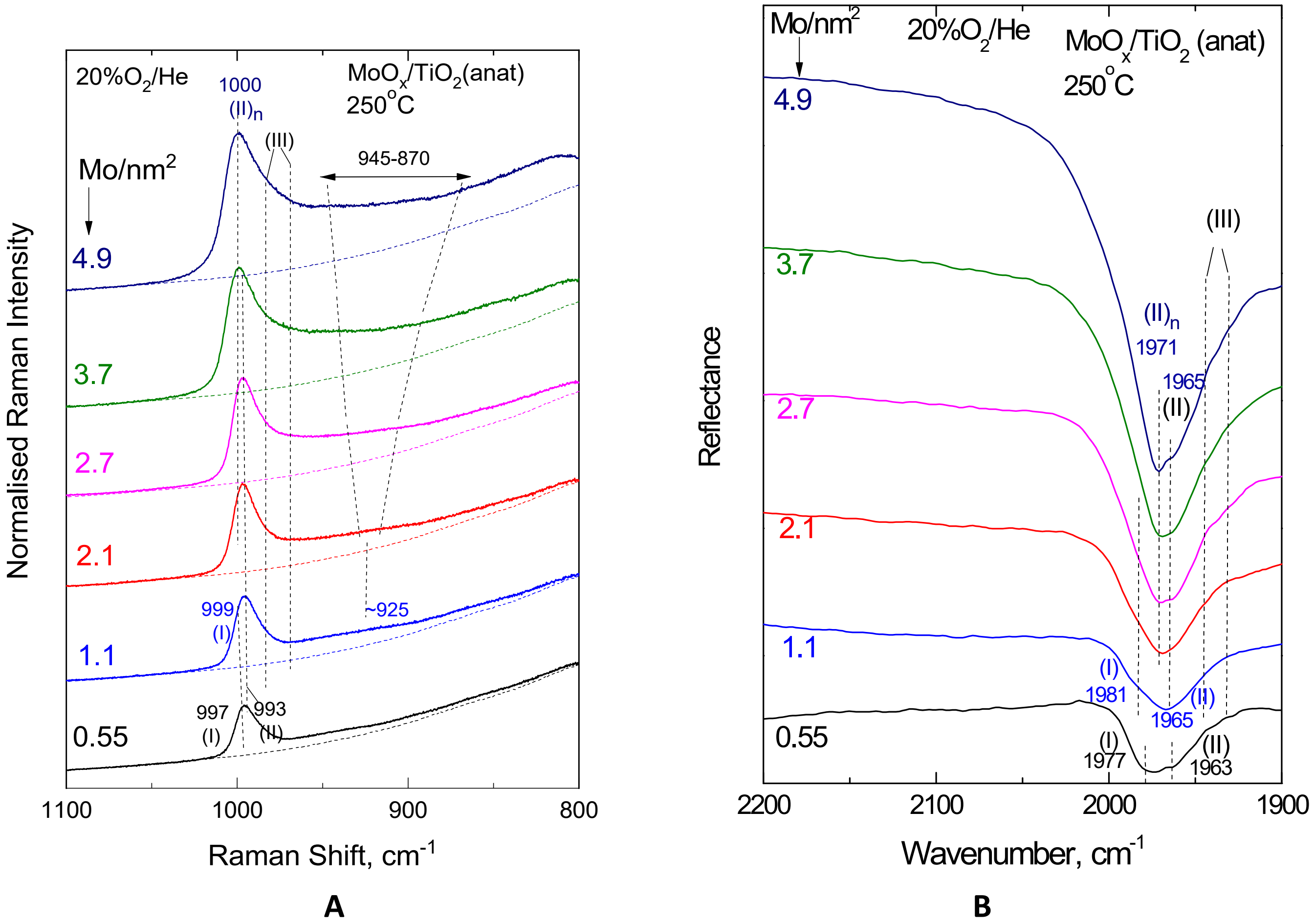

Figure S2. Effect of coverage for MoO_x/TiO_2(anatase) catalysts, in the 0.55 – 4.9 Mo/nm^2 range under flowing 20% O_2/He at 250 °C. (A) *in situ* Raman spectra. Recording parameters: see the caption to Fig. 2(A). (B) *in situ* FTIR spectra.

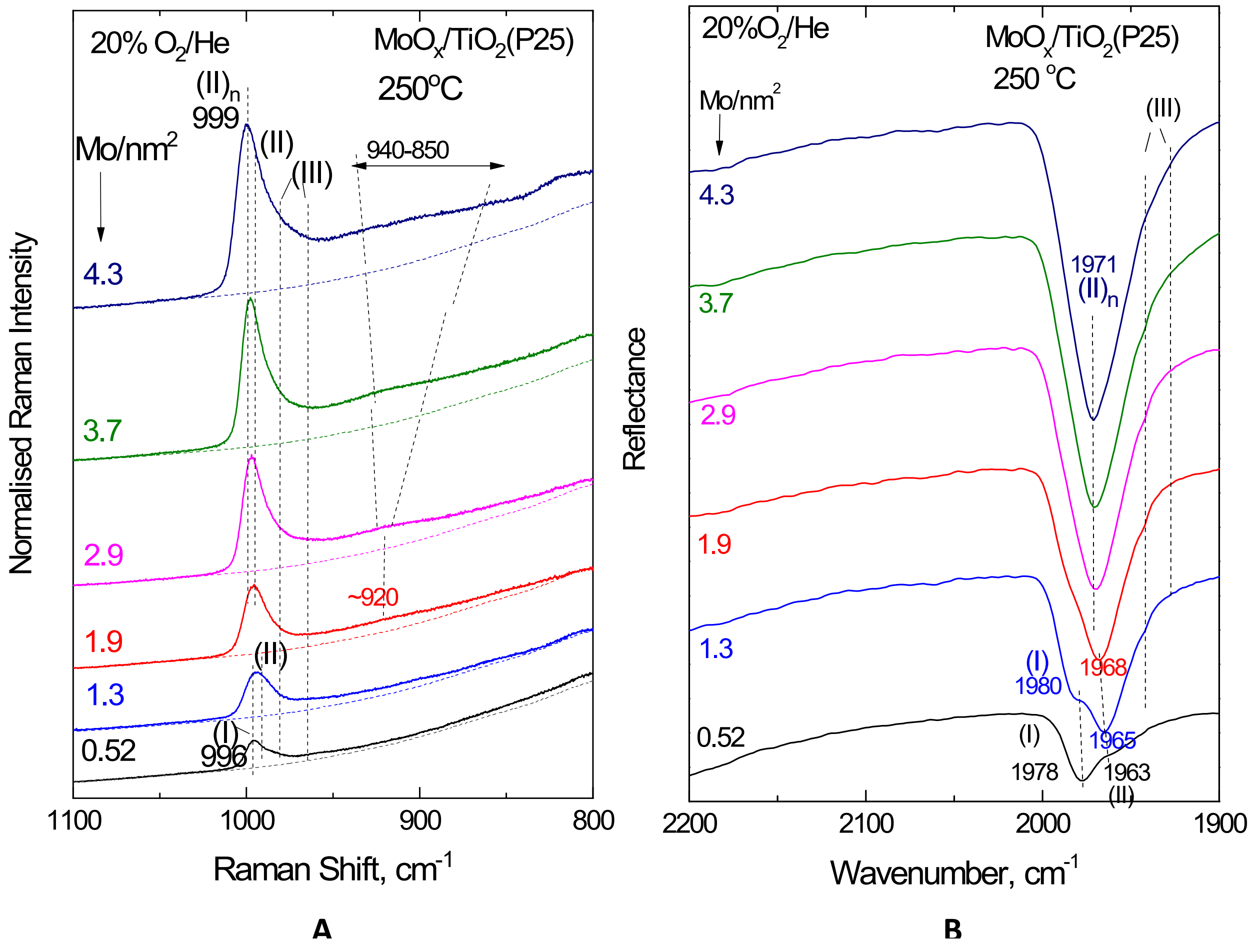

Figure S3. Effect of coverage for MoO_x/TiO_2(P25) catalysts, in the 0.55 – 4.9 Mo/nm^2 range under flowing 20% O_2/He at 250 ^oC. (A) *in situ* Raman spectra. Recording parameters: see the caption to Fig. 2(A). (B) *in situ* FTIR spectra.

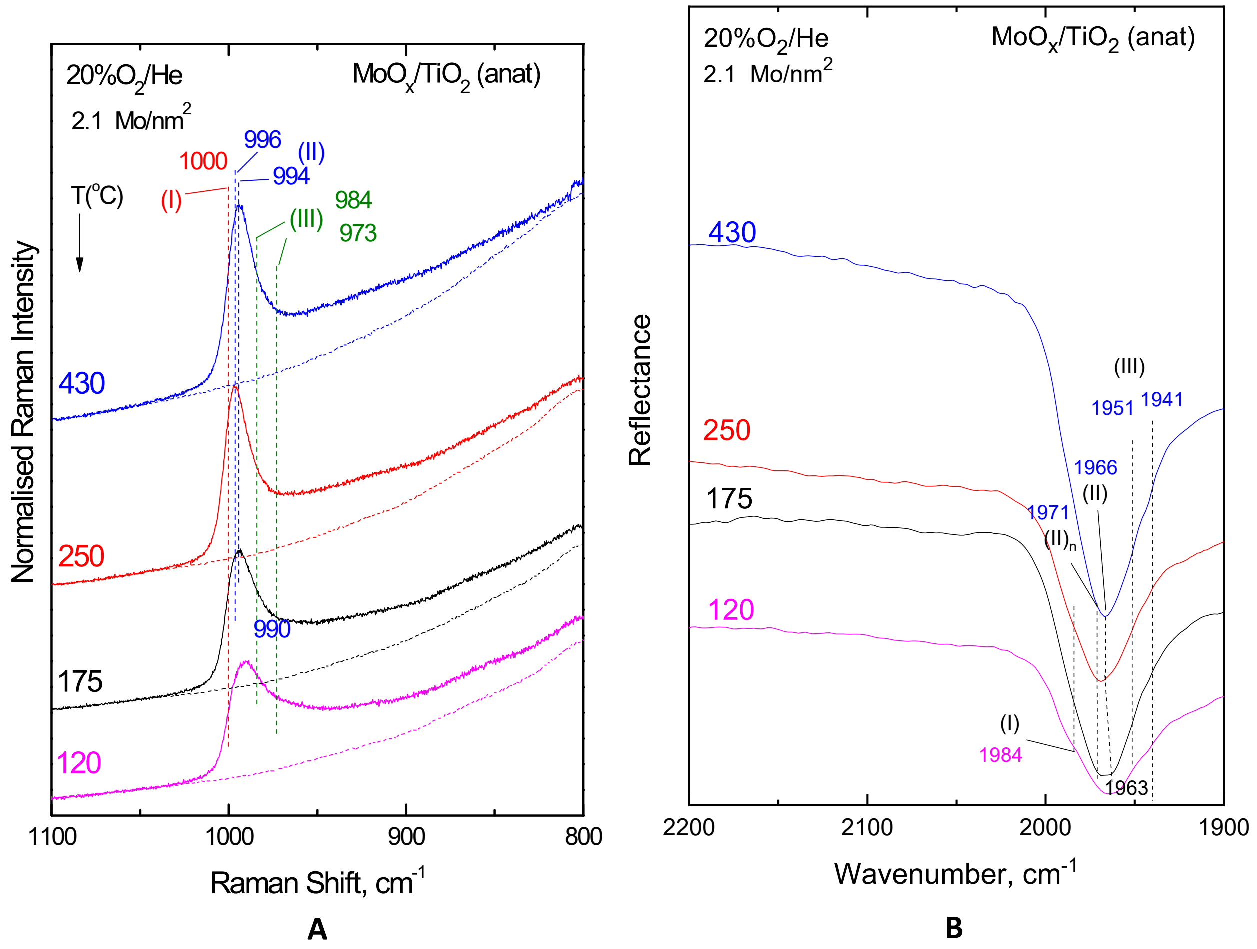

Figure S4. MoO_x/TiO_2(anatase) with surface density of 2.1 Mo/nm^2. (A) See caption to Fig. 2(A). (B) See caption to Figure 2(C).

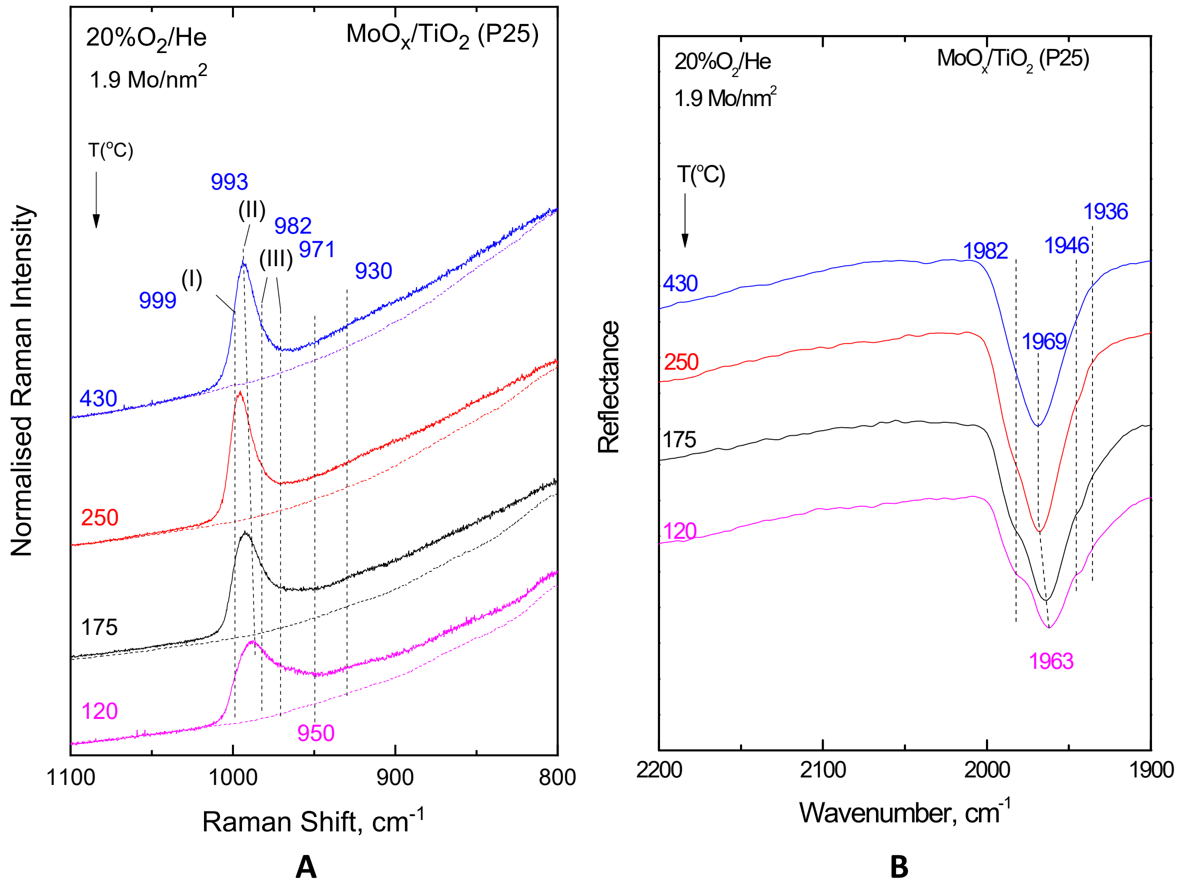

Figure S5. MoO_x/TiO_2(P25) with surface density of 1.9 Mo/nm^2. (A) See caption to Fig. 2(A). (B) See caption to Figure 2(C).

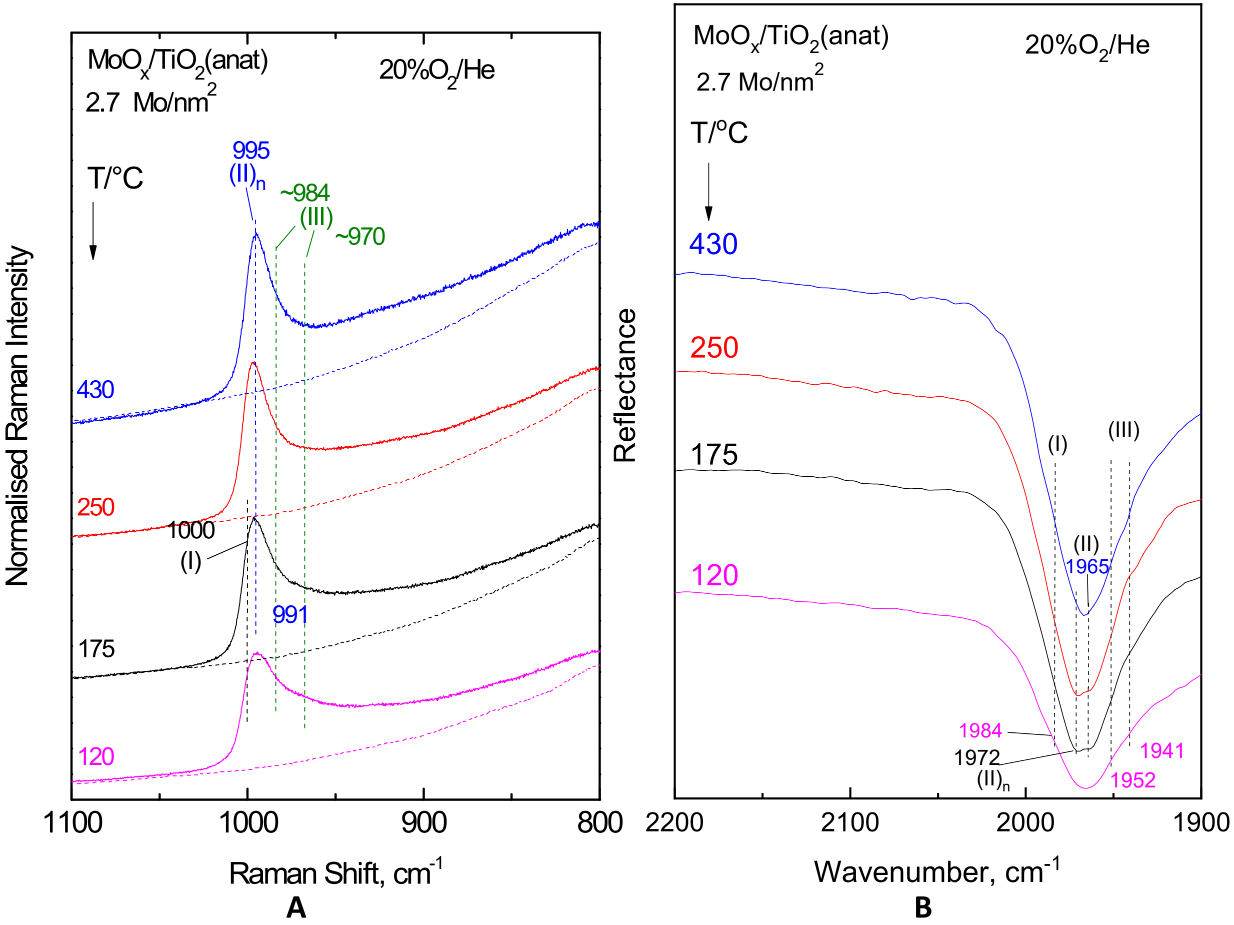

Figure S6. MoO_x/TiO_2(anatase) with surface density of 2.7 Mo/nm^2. (A) See caption to Fig. 2(A). (B) See caption to Figure 2(C).

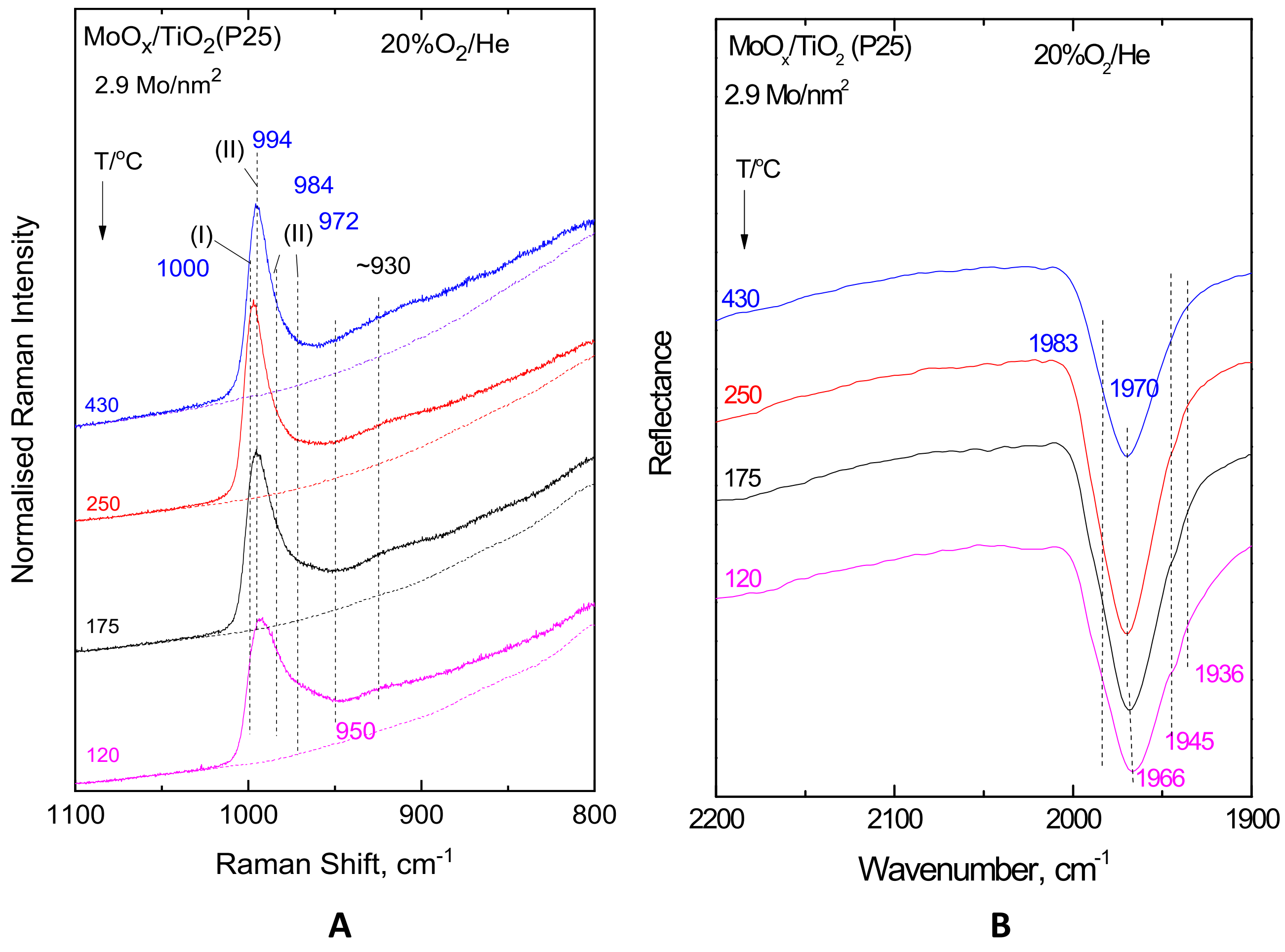

Figure S7. MoO_x/TiO_2(P25) with surface density of 2.9 Mo/nm^2. (A) See caption to Fig. 2(A). (B) See caption to Figure 2(C).

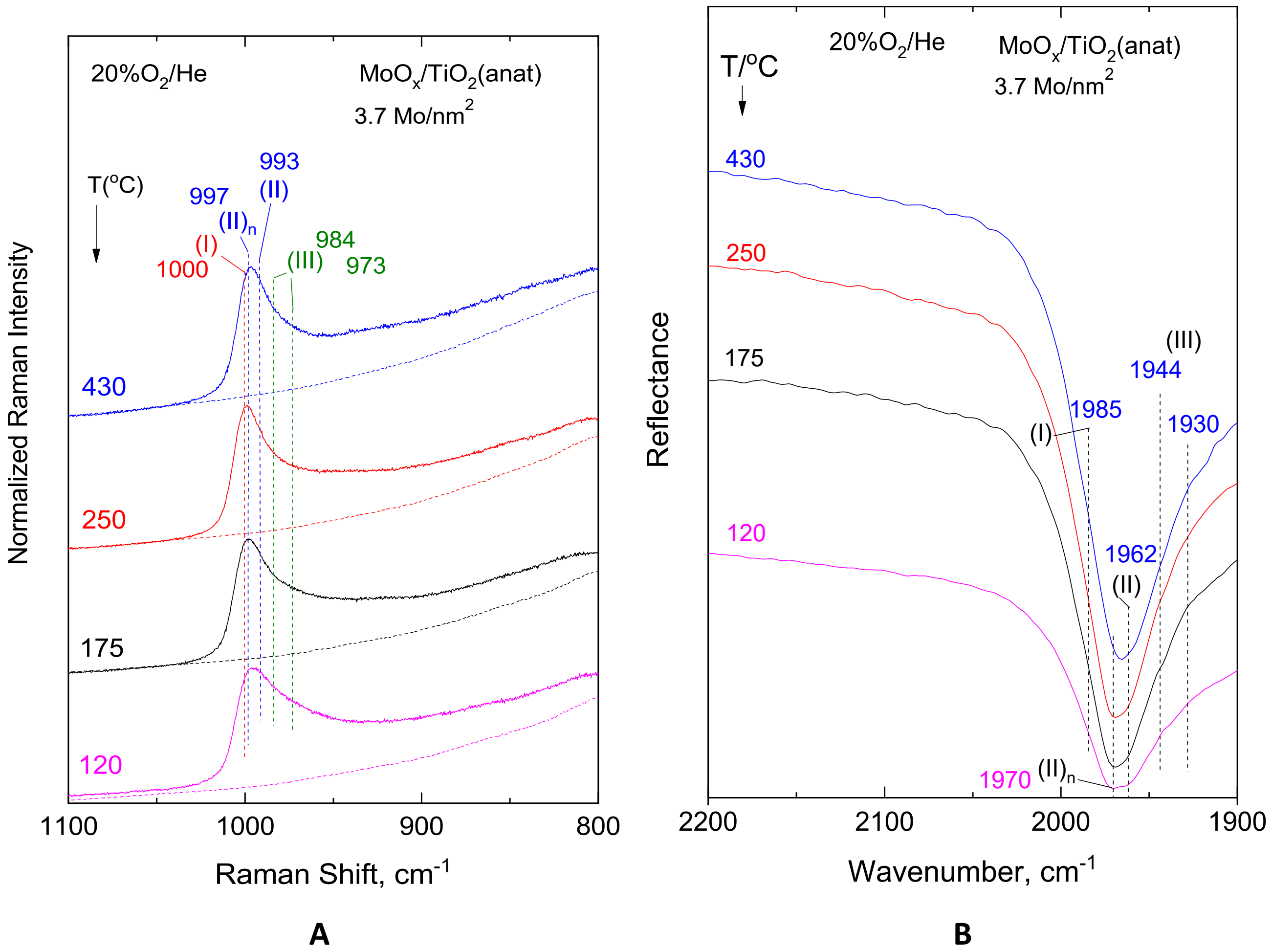

Figure S8. MoO_x/TiO_2(anatase) with surface density of 3.7 Mo/nm^2. (A) See caption to Fig. 2(A). (B) See caption to Figure 2(C).

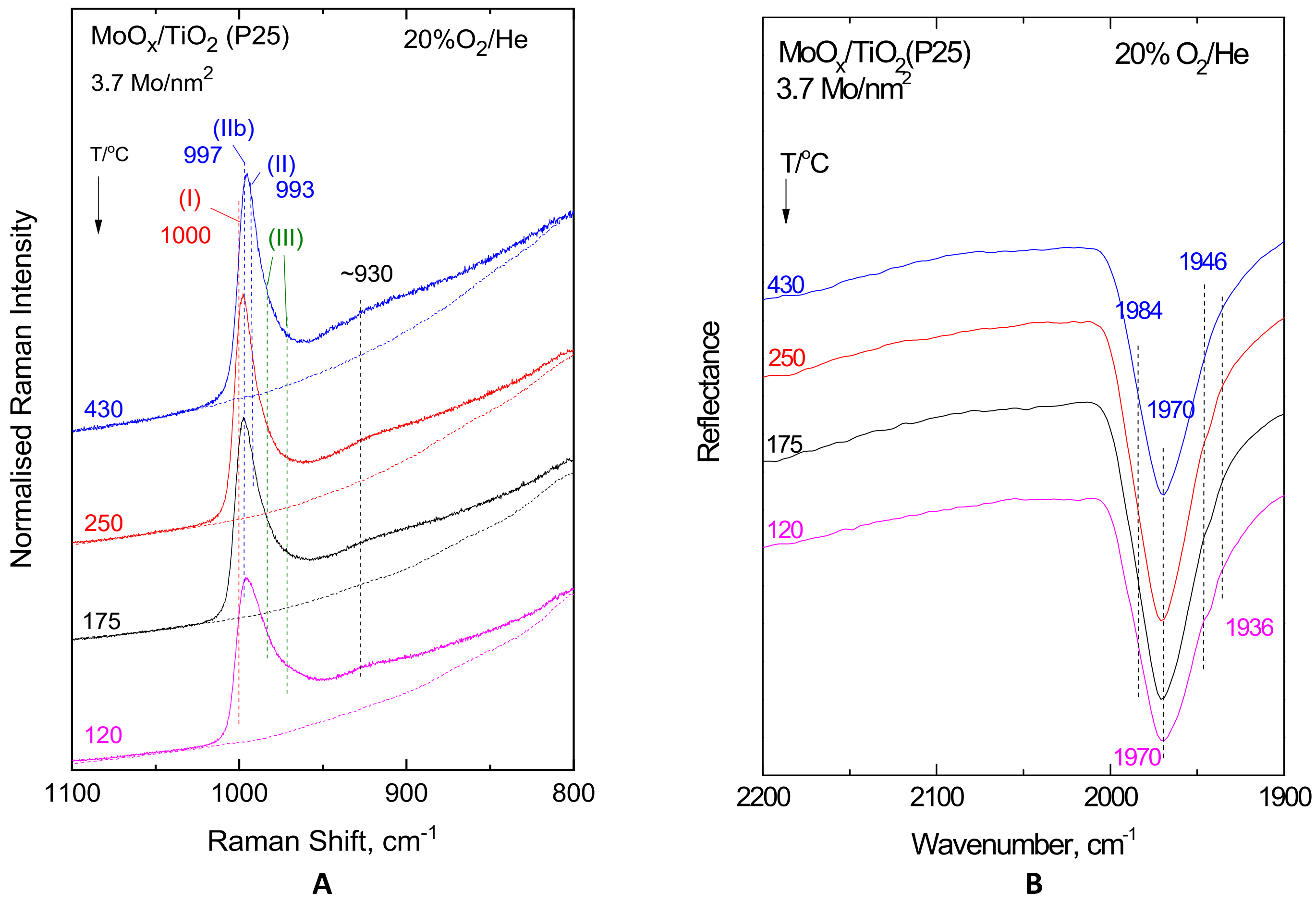

Figure S9. MoO_x/TiO_2(P25) with surface density of 3.7 Mo/nm^2. (A) See caption to Fig. 2(A). (B) See caption to Figure 2(C).

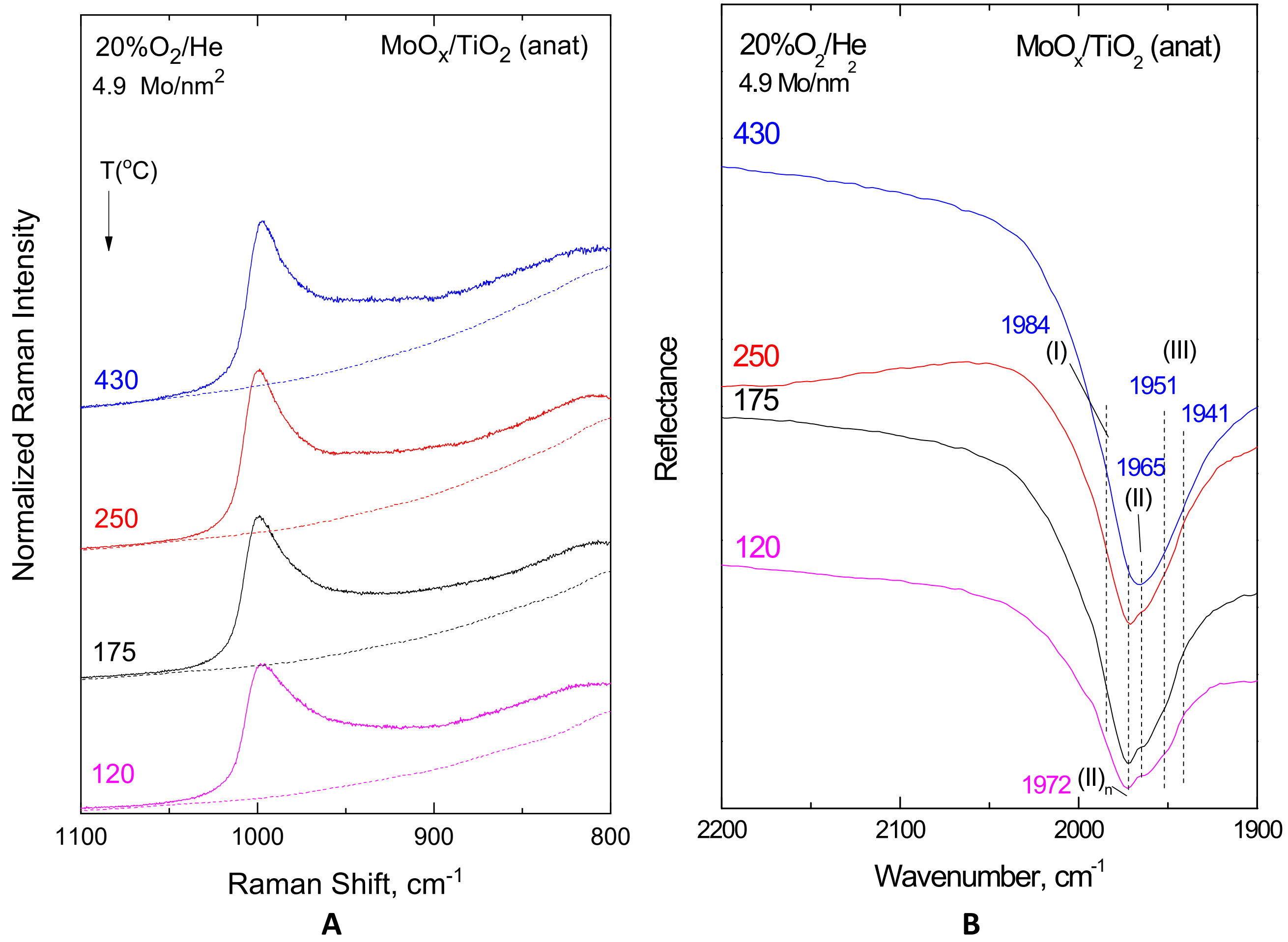

Figure S10. MoO_x/TiO_2(anatase) with surface density of 4.9 Mo/nm^2. (A) See caption to Fig. 2(A). (B) See caption to Figure 2(C).

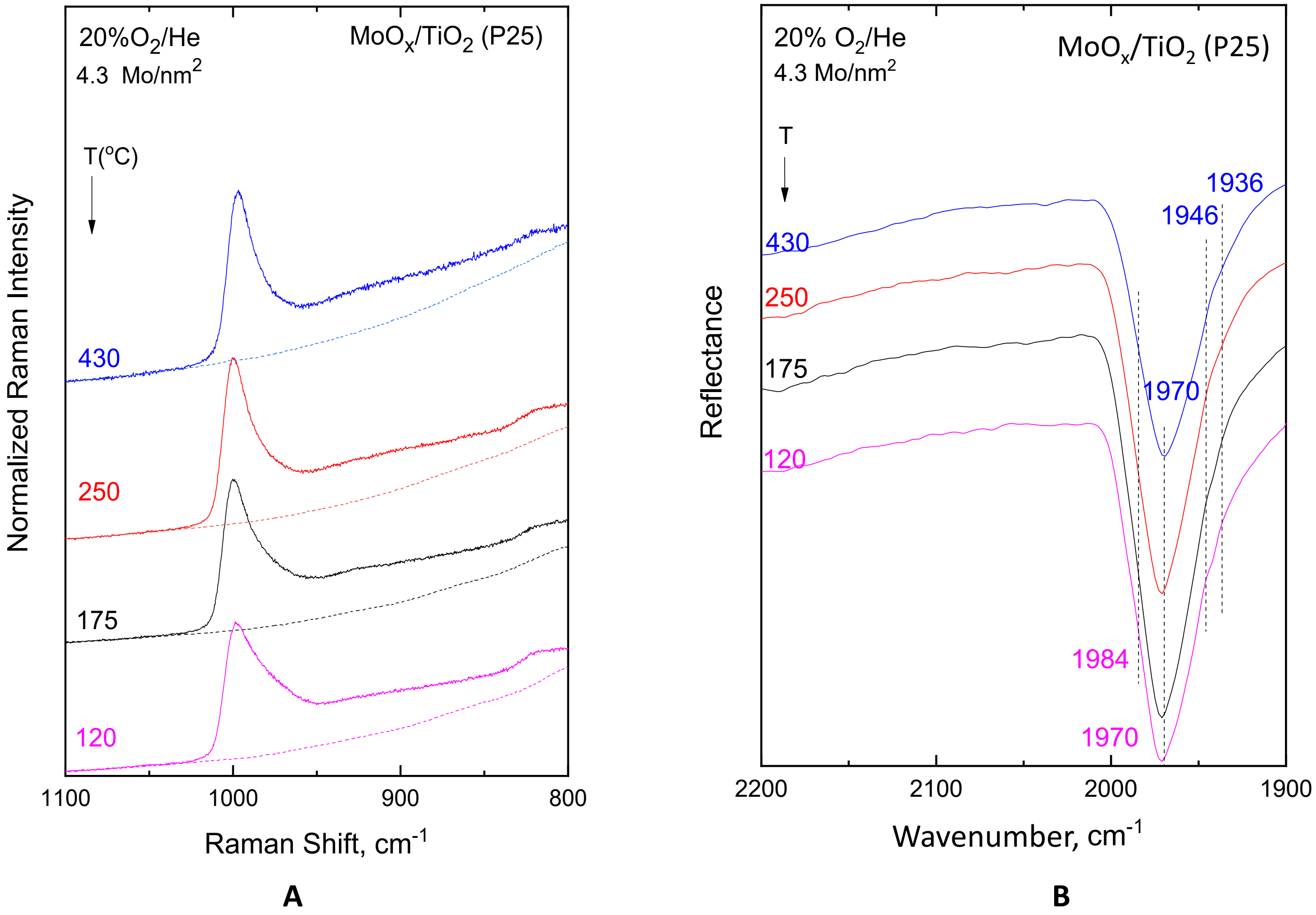

Figure S11. MoO_x/TiO_2(P25) with surface density of 4.3 Mo/nm^2. (A) See caption to Fig. 2(A). (B) See caption to Figure 2(C).
